# Sex differences in suicide, suicidal ideation, and self-harm after release from incarceration: a systematic review and meta-analysis

**DOI:** 10.1007/s00127-022-02390-z

**Published:** 2022-12-03

**Authors:** Emilia Janca, Claire Keen, Melissa Willoughby, Rohan Borschmann, Georgina Sutherland, Sohee Kwon, Stuart A. Kinner

**Affiliations:** 1 Curtin School of Population Health, Curtin University, 410 Koorliny Way, Bentley, WA 6102 Australia; 2 Justice Health Unit, Melbourne School of Population and Global Health, The University of Melbourne, 207 Bouverie Street, Carlton, VIC 3053 Australia; 3 Centre for Adolescent Health, Murdoch Children’s Research Institute, 50 Flemington Road, Parkville, VIC 3052 Australia; 4 Department of Psychiatry, University of Oxford, Warneford Hospital, Oxford, OX3 7JX UK; 5 Melbourne School of Psychological Sciences, The University of Melbourne, Grattan Street, Parkville, VIC Australia; 6 Disability and Health Unit, Melbourne School of Population and Global Health, The University of Melbourne, 207 Bouverie Street, Carlton, VIC 3053 Australia; 7 Mater Research Institute-UQ, University of Queensland, Mater Hospital, Raymond Terrace, South Brisbane, QLD 4101 Australia; 8 Griffith Criminology Institute, Griffith University, 176 Messines Ridge Road, Mount Gravatt, QLD 4122 Australia; 9 School of Public Health and Preventive Medicine, Monash University, 553 St Kilda Road, Melbourne, VIC 3004 Australia

**Keywords:** Self-harm, Suicide, Suicidal ideation, Sex differences, Incarceration, Systematic review

## Abstract

**Purpose:**

People released from incarceration are at increased risk of suicide compared to the general population. We aimed to synthesise evidence on the incidence of and sex differences in suicide, suicidal ideation, and self-harm after release from incarceration.

**Methods:**

We searched MEDLINE, EMBASE, PsycINFO, Web of Science and PubMed between 1 January 1970 and 14 October 2021 for suicide, suicidal ideation, and self-harm after release from incarceration (PROSPERO registration: CRD42020208885). We calculated pooled crude mortality rates (CMRs) and standardised mortality ratios (SMRs) for suicide, overall and by sex, using random-effects models. We calculated a pooled incidence rate ratio (IRR) comparing rates of suicide by sex.

**Results:**

Twenty-nine studies were included. The pooled suicide CMR per 100,000 person years was 114.5 (95%CI 97.0, 132.0, *I*^2^ = 99.2%) for non-sex stratified samples, 139.5 (95% CI 91.3, 187.8, *I*^2^ = 88.6%) for women, and 121.8 (95% CI 82.4, 161.2, *I*^2^ = 99.1%) for men. The suicide SMR was 7.4 (95% CI 5.4, 9.4, *I*^2^ = 98.3%) for non-sex stratified samples, 14.9 for women (95% CI 6.7, 23.1, *I*^2^ = 88.3%), and 4.6 for men (95% CI 1.3, 7.8, *I*^2^ = 98.8%). The pooled suicide IRR comparing women to men was 1.1 (95% CI 0.9, 1.4, *I*^2^ = 82.2%). No studies reporting self-harm or suicidal ideation after incarceration reported sex differences.

**Conclusion:**

People released from incarceration are greater than seven times more likely to die by suicide than the general population. The rate of suicide is higher after release than during incarceration, with the elevation in suicide risk (compared with the general population) three times higher for women than for men. Greater effort to prevent suicide after incarceration, particularly among women, is urgently needed.

**Supplementary Information:**

The online version contains supplementary material available at 10.1007/s00127-022-02390-z.

## Introduction

People in custody experience elevated rates of suicide, suicidal ideation, and self-harm compared to the general population [1–3]. Although suicide is more common among men than women in the general population, self-harm and suicidal ideation are more common among women [4, 5]. Research on suicide, suicidal ideation, and self-harm among people involved in the criminal justice system typically focuses on time during incarceration [1]. There is some evidence that rates of suicide [6], self-harm [7], and suicidal ideation [8] during incarceration are similar between sexes, in contrast with general population trends [4, 9].

People with a history of incarceration often experience major social challenges upon return to the community, including homelessness [10], substance use [11], and unemployment [12]. People with a history of incarceration also experience elevated rates of poor health and preventable death compared to the general population [13], including by suicide [14]. Women with a history of incarceration experience poorer health and social circumstances than both their male counterparts and women in the general population [15–17]. Exposure to trauma and abuse, and the consequences of poor mental health, substance use and homelessness, are all common among justice-involved women [18–20], and contribute to their markedly poor health profiles after release from incarceration.

A previous systematic review conducted in 2012 [21] that examined all-cause and external-cause deaths among people released from incarceration reported crude mortality rates (CMRs) for suicide ranging from 41 to 204 deaths per 100,000 person years among people released from incarceration [21]. This is substantially higher than the rate of 11 per 100,000 person years reported in the global general population [4]. However, this review did not calculate a pooled CMR or standardised mortality ratios (SMRs) for suicide. A meta-analysis conducted in 2013 and including five studies found that people released from incarceration were roughly seven times more likely to die by suicide than the general population (pooled risk ratio = 6.8, 95% CI 6.1, 7.5) [22]. Neither review examined differences in suicide between women and men after release from incarceration. To date, no reviews have looked at self-harm and/or suicidal ideation in women or men after release from incarceration.

As the number of people who experience incarceration continues to increase globally [23], robust and reliable estimates of suicide, self-harm, and suicidal ideation in people released from incarceration are needed to support and inform effective prevention strategies. Given the disproportionate growth in the number of women incarcerated globally [24], and their unique health and social needs [25], developing evidence-based and targeted interventions to reduce suicide, self-harm, and suicidal ideation among people released from incarceration requires an understanding of differences between women and men in this population. As such, we aimed to (1) calculate the incidence and risk relative to the general population of suicide, self-harm, and suicidal ideation among people released from incarceration, overall and stratified by sex; and (2) examine the association between sex and suicide, self-harm, and suicidal ideation.

## Methods

### Search strategy and selection criteria

Our review is reported in accordance with the Preferred Reporting Items for Systematic Reviews and Meta-analysis (PRISMA) guidelines (Table S1) [26, 27]. The review protocol was registered with the International Prospective Register of Systematic Reviews (PROSPERO; CRD42020208885), and was updated on 14 December 2021.

We searched five key health and social science databases (MEDLINE, EMBASE, PsycINFO, Web of Science, and PubMed) using search terms relating to incarceration and release, suicide, self-harm, and suicidal ideation for literature published from 1 January 1970 to 8 September 2020. The search strategy (Table S2) was developed in consultation with a trained research librarian. Reference lists of included studies were checked to identify any additional relevant studies not captured by the search. As done previously [28], and as described in PRISMA guidelines [26], we updated our search by receiving email alerts from the Web of Science Core Collection for records published between 9 September 2020 and 14 October 2021.

Studies were eligible for inclusion if they were published in peer-reviewed journals, reported on suicide, self-harm, and/or suicidal ideation occurring in the community following release from incarceration (including youth justice detention, prison, and jail), and reported at least one measure of interest or sufficient data for calculation (CMR, SMR, and/or an association between sex and any outcome). We contacted authors if the total number of participants (overall and/or sex-stratified) could not be determined from the study. Studies that reported CMRs and SMRs overall and/or stratified by sex were eligible for inclusion. The definitions of suicide, self-harm, and suicidal ideation used in this review are presented in Table S3.

Studies published in languages other than English were excluded. We included studies from all geographic locations. Previous literature reviews were excluded; however, the reference lists of these reviews were checked for additional relevant studies. Consistent with previous literature [21], studies reporting on selected samples (e.g., people who use drugs) were excluded.

After the removal of duplicates, titles and abstracts of potentially eligible studies were screened by EJ with MW also screening a random 10% sample. There was moderate inter-rater agreement between the two reviewers (kappa value 0.79) [29]. Any uncertainty related to study inclusion was resolved through discussion between the authors. Full-text articles were independently screened once by EJ and then again by MW, CK, or SoK. Any conflicts were resolved through discussion. We included studies that used the same study data where articles reported different findings of interest. In the case that multiple studies reported the same findings, both studies were included in the review, but only the study with the longest follow-up period was included in meta-analysis.

## Data analysis

Data were independently extracted by EJ (a summary of the information extracted is provided in Table S4). If not directly reported, we calculated CMRs and SMRs (and 95% CI) for suicide in a manner consistent with previous research [21]. Using standard formulae [30], for each study that reported a CMR for both women and men, we calculated incidence rate ratios (IRRs, and 95% CI) for suicide using men as the reference group.

Study quality was assessed using the Methodological Standard for Epidemiological Research (MASTER) scale [31]. The MASTER scale ranks studies based on the number of safeguards against bias present in the study, with a higher number of safeguards indicating a lower probability of bias (score range 0, 40) [31]. Risk of bias of each study was assessed by EJ and any uncertainty was resolved through discussion and consensus with CK (scores for each study are presented in Table 1).

**Table 1:** Characteristics of included studies

First author, year	City/state, country	Study type	Years of observation	Median follow up (years)	Outcome	Sex	Detention type	Age at baseline (years)	Participants *n* (%)			Reference population for SMR	QAS
									All	Men	Women		
Barnert, 2019 [66]	US (national)	R	1994–2008	NR	Suicidal ideation	Women & men	Youth detention	Range: 7–24	1727	1344 (77.8)	383 (22.2)	NA	18
Binswanger, 2007 [51]	Washington, US	R	1999–2003	NR	Suicide	Women & men	Prison	Mean: 33.4 ± 9.8 (Range: 18–84)	30,237	26,270 (87.0)	3697 (13.0)	Washington State residents of the same age, sex, and race	24
Binswanger, 2013 [52]	Washington, US	R	1999–2009	NR	Suicide	Women & men	Prison	Range: 18–84	76,208	63,979 (84.0)	12,229 (16.0)	Washington State residents of the same age, sex, and race	25
Bird, 2003 [39]^a^	Scotland	R	1996–1999	NR	Suicide	Men only	Prison	Range: 15–35	19,486	19,486 (100)	NA	15–35-year-old men in Scotland	22
Borschmann, 2017a [64]	Queensland, Australia	P	2007–2013	2.6	Self-harm	Women & men	Prison	Range: 18–40 +	1307	1030 (78.8)	277 (21.2)	NA	28
Borschmann, 2017b [65]	Queensland, Australia	P	2007–2014	3.9	Self-harm	Women & men	Prison	Range: 18–40 +	1309	976 (74.6)	260 (19.9)	NA	28
Brinkley-Rubinstein, 2019 [7]	North Carolina, US	R	2000–2015	NR	Suicide	Women & men	Prison	Median: 32 (IQR: 26–42)	229,274	197,656 (86.2)	31,618 (13.8)	NA	24
Bukten, 2017 [41]^b^	Norway (national)	P	2000–2014	NR	Suicide	Women & men	Prison	NR	92,663	(Range: 90.0–92.0)	(Range: 8.0–10.0)	NA	23
Bukten, 2021 [61]	Norway (national)	R	2000–2016	NR	Suicide	Women & men	Prison	Median: 31 (IQR: 23–41)	96,735^c^	86,761 (89.7)	9957 (10.3)	NA	26
Chang, 2015 [50]	Sweden (national)	R	2000–2009	5.1	Suicide	Women & men	Prison	NR	47,326	43,840 (92.6)	3486 (8.0)	NA	28
Coffey, 2003 [53]	Victoria, Australia	R	1988–1999	3.3 (men) 1.4 (women)	Suicide	Women & men	Youth detention	NR	2849	2621 (92.0)	228 (8.0)	Calendar year, sex, age stratified Victoria residents	22
Coffey, 2004 [54]	Victoria, Australia	R	1988–2003	6.3 (men) 4.6 (women)	Suicide	Women & men	Youth detention	NR	2849	2625 (92.0)	228 (8.0)	NA	23
Dirkzwager, 2012 [55]	Netherlands	R	1977–2003	NR	Suicide	Women & men	Prison	Range: 12–40 +	597	578 (96.8)	19 (3.2)	Age and gender adjusted 1977 Dutch population	22
Farrell, 2008 [62]	England and Wales (national)	R	1998–2003	NR	Suicide	Women & men	Prison	15 +	48,771	36,513 (74.9)	12,258 (25.1)	NA	19
Graham, 2003 [56]	Victoria, Australia	R	1990–2000	NR	Suicide	Women & men	Prison	NR	25,469	22,978 (90.2)	2490 (9.8)	Age, sex stratified Victorian residents	18
Haglund, 2014 [49]	Sweden (national)	P	2005–2009	1.2	Suicide	Women & men	Prison	Mean: 37.8 (SD: 12.1, range: 18–84)	38,995	36,146 (92.7)	2849 (7.3)	Gender and age matched Swedish people from general population (without incarceration history)	26
Harding-Pink, 1990 [63]^a^	Geneva, Switzerland	R	1982–1986	NR	Suicide	Women & men	Prison	NR	NR	NR	NR	NA	16
Jones, 2017 [58]	North Carolina, US	R	2008–2012	NR	Suicide	Women & men	Prison	Range: 20–60 +	41,495	37,053 (89.3)	4442 (10.7)	NA	19
Kariminia, 2007a, b [14, 42]	New South Wales, Australia	R	1988–2003	7.7	Suicide	Women & men	Prison	NR	85,203	76,383 (89.6)	8820 (10.4)	Age, sex stratified New South Wales population	22
Kouyoumdjian, 2016 [43]	Ontario, Canada	R	2000–2012	NR	Suicide	Women & men	Prison	NR	48,166	43,419 (90.1)	4747 (9.9)	General Canadian population	24
Lim, 2012 [44]	New York City, US	R	2001–2005	NR	Suicide	Women & men	Jail	Range: 16–89	155,272	137,161 (88.3)	18,111 (12.7)	Age matched New York City residents	21
Pratt, 2006 [48]	England and Wales	R	2000–2002	NR	Suicide	NR	Prison	Range: 18–50 +	244,988	NR	NR	Age, sex stratified “general populations”	21
Rosen, 2008 [59]	North Carolina, US	R	1980–2005	10.3	Suicide	Men only	Prison	Median: 32 (IQR:25–40, range: 20–69)	168,001	168,001 (100.0)	NA	Mid-years from 2008 to 2012 North Carolina population in residents matched on County, sex, race, and age	22
Rosen, 2020 [60]	North Carolina, US	R	2008–2016	NR	Suicide	Women & men	Prison	Range: 18–88	111,479	96,367 (86.4)	15,112 (13.6)	NA	20
Sailas, 2006 [57]	Finland (national)	R	1984–2002	11.7	Suicide	Women & men	Youth detention	Range: 15–21	3832	3743 (97.7)	89 (2.3)	NA	20
Spittal, 2014 [47]	Queensland, Australia	R	1994–2007	7.5	Suicide	Women & men	Prison	Range: 17–40 +	41,970	36,994 (88.1)	4976 (12.9)	Age and sex matched general population	22
Stewart, 2004 [45]	Western Australia, Australia	R	1994–1999	3.4	Suicide	Women & men	Prison	Mean: 31, median: 29, mode: 21 (range: 16–82)	9381	8199 (87.4)	1182 (13.6)	Age, sex, race matched Western Australian population	25
van Dooren, 2013 [46]	Queensland, Australia	R	1994–2007	NR	Suicide	Women & men	Prison	NR	42,015	37,039 (88.2)	4976 (11.8)	Age and sex matched Queensland general population	22

*NA* not applicable, *NR* not reported, *P* prospective, *R* retrospective, *US* United States, *QAS* Quality Assessment Score

^a^Included in the review but excluded from primary meta-analyses because there was either less than 6 months follow up time and/or fewer than 20 suicides

^b^Included in the review but excluded from primary meta-analyses because another study from the same cohort reported findings with a longer follow-up time (i.e., Bukten 2021)

^c^Refers to gender, and not sex. Gender was unknown for 17 participants

We calculated pooled estimates of CMRs and SMRs for death by suicide, overall and stratified by sex. Using the IRRs for suicide, with men as the reference group, we calculated a pooled estimate of the IRR for suicide according to sex. Estimates were pooled using the DerSimonian Laird method [32]. A random-effects method was used because we did not expect the assumptions of a fixed-effects model to be met (i.e., the assumption of a common effect size) [33]. Heterogeneity between studies was assessed using the *I*^2^ statistic [34].

We extracted data from studies reporting other measures of association (e.g., odds ratios or hazard ratios) between sex and suicide. Due to the small number of studies reporting other measures of association between sex and suicide, and the diversity of measures reported, it was not possible to pool other measures of associations between sex and suicide.

Given the limited number of studies, it was not possible to calculate pooled estimates for self-harm or suicidal ideation (overall or sex-stratified). The results for these outcomes are narratively described [35].

To examine the effect of study quality on the outcomes, we conducted a sensitivity analysis in which we restricted the analysis to studies scoring above the median on the MASTER scale. Due to the small number of studies that reported SMRs, we were only able to conduct this sensitivity analysis on the CMR and SMR meta-analyses that did not stratify by sex.

Where data were available, we conducted univariable meta-regression to identify factors which influenced the heterogeneity of effect measures. We did this using restricted maximum likelihood (REML) estimation with the Knapp–Hartung modification [36]. Values less than zero were rounded to zero. Consistent with previous research [37], the following factors were considered: type of incarceration facility (i.e., prison, jail, or youth detention), prospective/retrospective design, length of follow-up, geographic location of the study, single-sex samples, and whether subsequent periods of incarceration during follow-up were removed from analysis (i.e., interval censoring).

All analyses were conducted using Stata/BE Release 17 [38].

### Deviations from protocol

Consistent with previous reviews [21, 37], we modified our eligibility criteria by excluding studies from the primary analysis that had fewer than 20 total deaths from suicide or less than 6 months of follow-up. To test the effect of excluding these studies, we conducted a sensitivity analysis that included studies that did not meet these criteria but were otherwise eligible to be included in the review. As the studies that did not meet these criteria only reported non-sex stratified CMRs, we were only able to conduct this sensitivity analysis for the CMR not stratified by sex.

### Role of the funding source

There was no funding source for this study.

## Results

Our search retrieved 3284 records, 1711 of which remained after duplicates were removed (Figure S1). During title and abstract screening, 1556 records were excluded, leaving 155 full texts to be assessed. Of these, 27 met the eligibility criteria, along with an additional two records identified through citation searching. A total of 29 records were assessed for quality and included in this review.

The characteristics of included studies and characteristics of included participants are outlined in Table 1. The number of suicide deaths, person-years, CMRs, and SMRs for each study are presented in Table 2. Of the 29 included studies, there were 26 studies on suicide from 22 cohorts [14, 39–63], two studies on self-harm from one cohort [64, 65], and one study on suicidal ideation [66]. Data from 23 studies were included in meta-analyses for suicide. Two studies were only included in sensitivity analyses for suicide because they reported less than six months follow up time and/or fewer than 20 suicides. One study was not included in meta-analyses for suicide because it reported on a cohort for which longer follow-up time was available in another included study.

**Table 2:** Number of suicide deaths, time at risk following release (person-years), CMRs and SMRs for suicide deaths after release from prison, overall and stratified by sex

First author, year	*N* of suicide deaths			Person-years			Suicide death CMR (95% CI) per 100,000 person years			Suicide death SMR (95% CI)		
	Total	Women	Men	Total	Women	Men	Total	Women	Men	Total	Women	Men
Binswanger, 2007 [51]	40	NR	NR	57,049	NR	NR	18.0 (9.0–33.0)	NR	NR	3.4 (2.5–4.7)	NR	NR
Binswanger, 2013 [52]	212	21	191	334,238	NR	NR	63.0 (55.0–72.0)	68.0 (58.0–77.0)	41.0 (23.0–58.0)	3.2 (2.9–3.6)	NR	NR
Bird, 2003 [39]	10	NA	10	3797	NA	3797	263.4 (100.1–426.6)^a^	NA	NR	NR	NA	NR
Brinkley-Rubinstein, 2019 [40]	635	NR	NR	1,974,823	NR	NR	32.2 (29.7–34.7)^a^	NR	NR	NR	NR	NR
Bukten, 2017 [41]	74	NR	NR	NR	NR	NR	100.0 (80.0–130.0)	NR	NR	NR	NR	NR
Bukten, 2021 [61]	749	NR	NR	904,331	NR	NR	82.8 (76.9–88.8)	NR	NR	NR	NR	NR
Chang, 2015 [50]	471	36	435	238,457	16,935	221,522	198.0 (180.0–215.0)	213.0 (143.0–282.0)	196.0 (178.0–215.0)	NR	NR	NR
Coffey, 2003 [53]	23	1	22	11,333	619	10,714	NR	NR	NR	9.2 (5.8, 14.7)	NR	NR
Coffey, 2004 [54]	34	NR	NR	19,949	NR	NR	170.0 (120.0–240.0)	NR	NR	NR	NR	NR
Dirkzwager, 2012 [55]	12	NR	NR	NR	NR	NR	NR	NR	NR	6.7 (2.9–10.5)	NR	NR
Farrell, 2008 [62]^c^	36	NR	NR	48,578	NR	NR	74.0 (50.0–98.0)	NR	NR	NR	NR	NR
Graham, 2003 [56]	279	NR	NR	25,469	NR	NR	181.4 (160.1–202.7)^a^	NR	NR	NR	NR	NR
Haglund, 2014 [49]	127	12	115	NR	NR	NR	204.0 (168.5–239.5)^b^	NR	NR	18.4 (13.9–23.8)	NR	NR
Harding-Pink, 1990 [63]	5	NR	NR	8200	NR	NR	61.0 (7.5–114.4)^a^	NR	NR	NR	NR	NR
Jones, 2017 [58]	39	NR	NR	147,782	NR	NR	26.4 (18.1–34.7)^a^	NR	NR	14.5 (10.3–19.8)	NR	NR
Kariminia, 2007a, b [14, 52]^d^	724	46	678	557,352	56,354	500,998	129.9 (120.4–139.4)^a^	135 (96.0–174.0)^b^	82 (75.8, 88.2)^b^	4.2 (0.2–8.2)^a,d^	11.5 (4.9–18.2)^a,d^	4.0 (0.1–8.0)^a,d^
Kouyoumdjian, 2016 [43]	340	NR	NR	580,003	57,261	522,742	58.6 (52.4–64.9)^a^	NR	NR	4.3 (3.9–4.8)	NR	NR
Lim, 2012 [44]	35	5	30	379,363	NR	NR	9.2 (6.2–12.3)^a^	NR	NR	1.0 (0.7–1.4)	3.5 (1.2–8.3)	0.9 (0.6–1.2)
Pratt et al. 2006 [48]	382	34	348	244,988	18,942	226,046	156.0 (140.4–171.6)^b^	180.0 (119.5–240.5)^b^	154.0 (137.8–170.1)^a^	13.5 (12.2–14.9)	35.8 (25.4–50.2)	8.3 (7.5–9.3)
Rosen et al. 2008 [59]	746	NA	746	1,822,869	NA	1,822,869	40.9 (38.0–43.9)^a^	NA	40.9 (38.0–43.9)^a^	NR	NA	NR
Rosen et al. 2020 [60]	179	NR	NR	471,282	NR	NR	38.0 (32.4–43.6)^b^	NR	NR	NR	NR	NR
Sailas et al. 2006 [57]	148	NR	NR	43,411	NR	NR	340.9 (286.0–395.9)^a^	NR	NR	NR	NR	NR
Spittal et al. 2014 [47]	371	30	341	270,394	31,134	239,260	137.0 (123.1–151.0)^b^	96.0 (6.6–130.4)^b^	143.0 (127.8–158.2)^b^	7.6 (6.8–8.4)	14.2 (9.6–20.3)	4.8 (4.3–5.4)
Stewart et al. 2004 [45]	64	4	60	NR	NR	NR	203.7 (153.8–253.5)	NR	NR	5.0 (0.7–9.3)	16.1 (8.3–24.0)	4.7 (0.4–8.9)
van Dooren et al. 2013 [46]	84	11	73	38,769	4717	34,052	216.7 (170.3–263.0)^a^	233.2 (95.4–371.0)^a^	214.4 (165.2–263.6)^a^	NR	NR	NR

*CMR* crude mortality rate, *SMR* standardised mortality ratio, *NA* not applicable, *NR* not reported

^a^Point estimate and confidence interval calculated from available data

^b^Confidence interval calculated from available data

^c^Figures reported are those from Zlodre & Fazel (2012)

^d^Two studies from one cohort were included. We used data from Kariminia 2007b to calculate CMRs overall and by sex. For SMR calculations, we used the number of expected deaths for the general population from Kariminia 2007a and the number of observed deaths in the study cohort from Kariminia 2007b to calculate SMRs excluding time in prison

The data sources and outcome definitions used in each included study are summarised in Table S5. Of the 26 studies reporting on suicide, most (*n* = 23) reported using the International Classification of Diseases (ICD) to define death by suicide. Of the 26 suicide studies, 11 examined confirmed deaths by suicide exclusively (e.g., ICD9: E950, E959, ICD10: X60, X84) [14, 39–47, 61], three included both suicide and unnatural deaths with undetermined intent (e.g., ICD10: X60, X84, Y10, Y34) [48–50], and 12 did not provide detailed information about suicide definitions [51–60, 62, 63].

The 26 included studies that reported on suicide were published between 1990 and 2021, and had a median follow-up of 10 years (IQR: 6, 16 years). The median MASTER scale score was 21 (range 16, 26). Sources of bias included short follow up periods, and limitations regarding sampling (e.g., only including people who were incarcerated for their first offence). Most of the 26 suicide studies reported on non-sex stratified samples (*n* = 24), with all samples either mostly, or exclusively, comprising men. Two of the 26 suicide studies reported on male-only cohorts, and no studies reported on female-only cohorts. All 26 suicide studies reported on cohort studies, with 24 using a retrospective and two using a prospective design. Twenty-three studies reported on adult samples released from jail or prison, and three studies (with two from one cohort) reported on people released from youth detention. All studies included in this review (*n* = 29) were from high-income countries, most frequently Australia (*n* = 10). Reported sociodemographic characteristics of study participants are presented in Table S6.

### Suicide

**Fig. 1:**
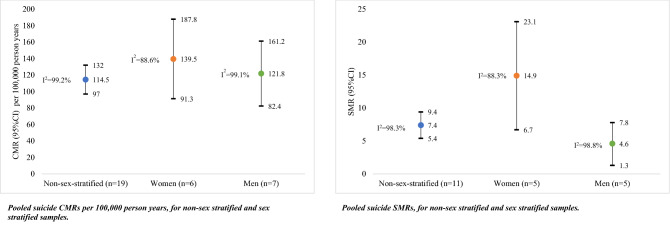
Pooled suicide CMRs per 100,000 person years, and pooled suicide SMRs, for non-sex stratified samples and stratified by sex. *n* = number of studies. This figure presents two stock plots. The first stock plot presents pooled suicide CMRs per 100,000 person years, for non-sex stratified samples, and stratified by sex. The pooled CMR for non-sex stratified samples was 114.5 per 100,000 person years (95% CI 97, 132, *I*^2^ = 99.2%). The pooled CMR for females was 139.5 per 100,000 person years (95% CI 91.3, 187.8, *I*^2^ = 88.6%). The pooled CMR for males was 121.8 per 100,000 person years (95% CI 82.4, 161.2, *I*^2^ = 99.1%). The suicide SMR was 7.4 (95% CI 5.4, 9.4, *I*^2^ = 98.3%) for non-sex stratified samples, 14.9 for women (95% CI 6.7, 23.1, *I*^2^ = 88.3%), and 4.6 for men (95% CI 1.3, 7.8, *I*^2^ = 98.8%)

Suicide CMRs for non-sex stratified samples were available in 19 studies. Suicide CMRs for women were reported in six studies, and for men in seven studies (including one male-only cohort). CMRs for suicide per 100,000 person years ranged from 9.2 to 340.9 for non-sex stratified samples, 68.0 to 233.2 for women, and from 40.9 to 214.4 for men. The pooled suicide CMR per 100,000 person years was 114.5 (95%CI 97.0, 132.0, *I*^2^ = 99.2%) for non-sex stratified samples, 139.5 (95% CI 91.3, 187.8, *I*^2^ = 88.6%) for women, and 121.8 (95%CI 82.4, 161.2, *I*^2^ = 99.1%) for men (Fig. 1). Forest plots for each estimate are presented in Figures S2–4. For all three pooled estimates, between-study heterogeneity was high (i.e., *I*^2^ > 75.0%), and Cochran’s Q tests were significant (i.e., *p* < 0.001).

We used univariable meta-regression to identify possible sources of heterogeneity in the pooled suicide CMR for non-sex stratified samples (Table 3). The pooled suicide CMR was higher for studies examining people released from youth detention compared to prison or jail (*p* = 0.03; *I*^2^ = 99.0%). Pooled suicide CMRs also varied significantly by country (*p* < 0.001; *I*^2^ = 96.7%; Table 3). We were not able to perform meta-regression for studies reporting female-only or male-only findings, due to the small number of studies that reported suicide CMRs by sex (*n* = 6 and *n* = 7 for women and men, respectively).

**Table 3:** Univariable meta-regressions of (1) the crude mortality rate (CMR) for suicide and of (2) the standarised mortality ratio (SMR) for suicide, by study factors

Factor	Meta-regression (1) Crude mortality rate (CMR) of suicide by study factors				Meta-regression (2) Standardised mortality ratio (SMR) of suicide by study factors			
	Number of studies (*n* = 19)	CMR (95% CI) per 100,000 person years	*p* value	*I*^2^	Number of studies (*n* = 11)	SMR (95% CI)	*p* value	*I*^2^
Type of incarceration facility								
Jail	1	9.2 (0.0, 163.2)	0.033	99.0%	1	1.0 (0.0, 12.7)	0.416	97.5%
Prison	16	113.5 (74.5, 152.4)			9	8.4 (4.3, 12.5)		
Youth detention	2	256.5 (139.0, 374.0)			1	9.2 (0.0, 22.1)		
Study design								
Prospective	1	204.0 (19.2, 388.8)	0.350	99.2%	1	6.7 (3.6, 9.9)	0.047	98.0%
Retrospective	18	117.5 (73.4, 160.7)			10	18.4 (7.4, 29.4)		
Interval censoring								
No	7	140.7 (70.2, 211.1)	0.494	99.2%	4	11.0 (5.4, 16.7)	0.131	97.3%
Yes	12	111.4 (58.0, 164.8)			7	5.9 (1.8, 10.0)		
Total length of follow-up (years)^a^								
≤ 10 years	9	123.4 (60.6, 186.1)	0.955	99.2%	6	10.1 (4.9, 15.3)	0.195	98.1%
> 10 years	10	121.0 (61.6, 180.5)			5	5.8 (1.14, 11.9)		
Country^b^								
Australia	6	166.5 (137.8, 195.1)	< 0.001	96.7%	4	6.6 (0.3, 12.8)	0.337	95.1%
Canada	1	58.6 (0.0, 118.6)			1	4.3 (0.0, 15.8)		
Finland	1	340.9 (255.4, 426.5)			–	–		
Netherlands	–	–	–	–	1	6.7 (0.0, 19.3)		
Norway	1	82.8 (22.9, 142.8)			–	–		
Sweden	2	200.6 (153.4, 247.8)			1	18.4 (5.1, 31.7)		
England and Wales	2	117.0 (72.1, 162.1)			1	13.5 (1.9, 25.1)		
US	6	34.9 (10.4, 59.3)			3	5.5 (0.0, 12.5)		
Male only samples^b^								
No	18	126.6 (83.5, 169.8)	0.344	99.24%	–	–	–	–
Yes	1	40.9 (0.0, 221.6)			–	–		

*CMR* crude mortality rate, *SMR* standardised mortality ratio, *95% CI* 95% confidence interval

^a^Included studies for meta-analysis 1 had a median follow up length of 10 years, range 0–25 years. Included studies for meta-analysis 2 had a median follow-up length of 10.8 years, range 0–25 years

^b^As no studies included in the primary analysis reported SMRs for men only, the “Male only samples” variable was not included in meta-regression 2

**Fig. 2:**
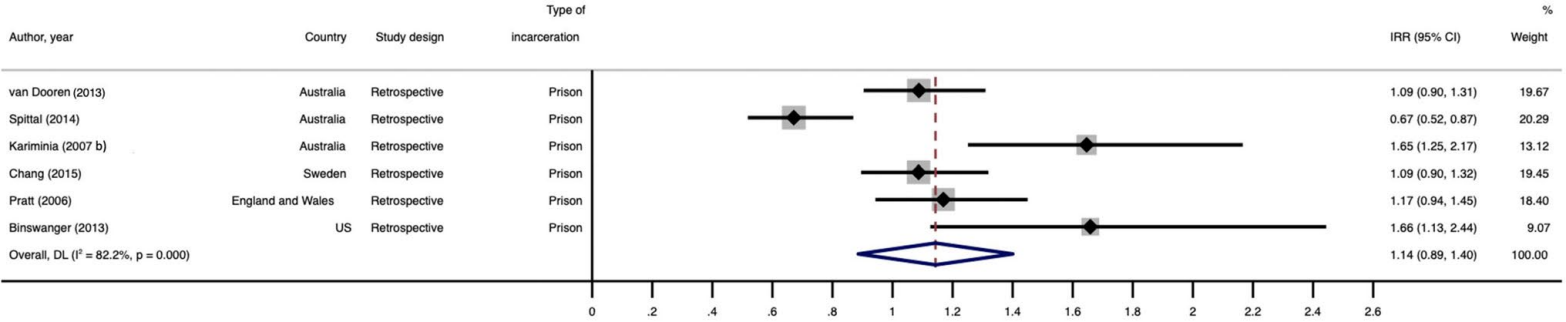
Forest plot for pooled IRR for suicide after release from prison comparing women and men. IRRs greater than 1.0 indicate a greater risk of suicide for women than men. This figure presents a forest plot for the pooled IRR for suicide after release from prison, comparing women and men. Six figures were included in this meta-analysis. The authors, figures and study characteristics are as follows: van Dooren [2013] (IRR = 1.09, 95% CI 0.90, 1.31; Australia); Spittal [2014] (IRR = 0.67, 95% CI 0.52, 0.87; Australia); Kariminia [2007b] (IRR = 1.65, 95% CI 1.25, 2.17; Australia); Chang (IRR = 1.09, 95% CI 0.90, 1.32; Sweden); Pratt [2006] (IRR = 1.17, 95% CI 0.94, 1.45; England and Wales); and Binswanger [2013] (IRR = 1.66, 95% CI 1.13, 2.44; US). All included studies used a retrospective design. The pooled IRR was 1.14 (95% CI 0.89, 1.40), and the *I*^2^ estimate was 82.2%

Six studies provided sufficient data to calculate suicide IRRs after release from incarceration by sex, using men as the reference group (Fig. 2). Suicide IRRs ranged from 0.7 to 1.8. The pooled suicide IRR estimate for the association between sex and suicide provided no indication of a difference in suicide risk between women and men (1.1, 95% CI 0.9, 1.4) The estimate of heterogeneity was high (*I*^2^ = 82.2%) and the Cochran’s Q test was significant (*p* < 0.001). Due to the small number of studies reporting suicide IRRs, we were unable to perform a meta-regression for this estimate.

**Fig. 3:**
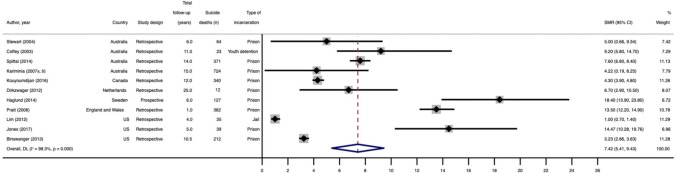
Forest plot for pooled suicide SMR for non-sex stratified samples. This figure presents a forest plot for the pooled SMR for suicide after release from prison, not stratified by sex. Eleven studies were included in this meta-analysis. The authors, and figures are as follows: Stewart [2004] (SMR = 5.00, 95% CI 0.66, 9.34; Australia); Coffey [2003] (SMR = 9.20, 95% CI 5.80, 14.70; Australia); Spittal [2014] (SMR = 7.60, 95% CI 6.80, 8.40); Kariminia [2007 a, b]; (SMR = 4.22, 95% CI 0.19, 8.25; Australia); Kouyoumdijan [2016] (SMR = 4.30, 95% CI 4.20, 95% CI 3.90, 4.80; Canada); Dirkzwager [2012] (SMR = 6.70, 95% CI 2.90, 10.50; Netherlands); Haglund [2014] (SMR = 18.40, 95% CI 13.90, 23.80; Sweden); Pratt [2006] (SMR = 13.50, 95% CI 12.20, 14.90; England and Wales); Lim [2012] (SMR = 1.00, 95% CI 0.70, 1.40; US); Jones [2017] (SMR = 14.47, 95% CI 10.28, 19.76; US); Binswanger [2013] (SMR = 3.23, 95% CI 2.86, 3.63; US). The pooled SMR was 7.42 (95% CI 5.41, 9.43), and the I^2^ estimate was 98.3%

Eleven studies reported suicide SMRs for non-sex stratified samples. Five studies reported suicide SMRs for women only and men only, respectively. The reference populations for suicide SMRs were usually the general population of the geographic location of the study matched on age, sex and/or ethnicity (Table 1). Suicide SMRs ranged from 1.0 to 18.4 for non-sex stratified samples, from 3.5 to 35.8 for women, and from 0.9 to 8.3 for men. The pooled suicide SMR for non-sex stratified samples was 7.4 (95% CI 5.4, 9.4, *I*^2^ = 98.3%). The pooled suicide SMR for women (14.9, 95% CI 6.7, 23.1, I^2^ = 88.3%) was more than three times greater than the pooled suicide SMR for men (4.6, 95% CI 1.3, 7.8, *I*^2^ = 98.8%). Forest plots for each estimate are presented in Fig. 3, and Figures S5 and 6. The pooled estimates are presented in Fig. 1. For all three pooled estimates, between-study heterogeneity was high (*I*^2^ > 75.0%), and the Cochran’s *Q* tests were significant (i.e., *p* < 0.001).

We used univariable meta-regression to identify possible sources of heterogeneity in the pooled suicide SMR for non-sex stratified samples (Table 3). The pooled suicide SMR was higher for studies with a retrospective compared to a prospective design (*p* < 0.05; *I*^2^ = 98.4%). We were not able perform meta-regressions for studies reporting female-only or male-only findings, due to the small number of studies that reported suicide SMRs by sex (*n* = 5 for women and *n* = 5 for men, respectively).

Six studies examined sex as a risk factor for suicide as part of their analyses (Table S7). No studies observed sex differences in the risk of suicide after release from incarceration.

The results of sensitivity analyses including studies that reported the measures of interest but did not meet the criteria to be included in the primary analysis (i.e., reporting fewer than 20 suicide deaths or less than six months of follow-up), were consistent with the results of the primary analysis (Tables S8–9; Figures S7, 9). The results of sensitivity analyses that restricted the analysis to studies with a score above the median on the MASTER scale were also consistent with the results of the primary analysis (Table S8, 10–11; Figures S8, 10) except that type of incarceration facility was no longer significant in the meta-regression for non-sex stratified CMRs (Table S10).

### Suicidal ideation and self-harm

The one study that reported on suicidal ideation after release from incarceration used data from 1727 participants of the US National Longitudinal Study of Adolescent to Adult Health. Data on incarceration history were collected during Wave I of the study (in 1994) and self-reported data on suicidal ideation were collected during Wave IV (in 2008). This study examined sex as a risk factor for adult suicidal ideation and found no difference between women and men (adjusted odds ratio 1.2, 95% CI 0.7, 2.2; male reference group).

The two included studies on self-harm after release from incarceration were from the same cohort, comprising 277 women and 1030 men released from prisons in Queensland, Australia. This cohort was followed prospectively using linked administrative health records. The first study reported a higher incidence of self-harm-related emergency department presentations for women released from incarceration (IR = 60.5 per 1000 person-years) compared to their male counterparts (IR = 49.2 per 1000 person-years). The second study used ambulance records to determine the incidence of ambulance attendances due to self-harm in the cohort. Rates of ambulance attendance for self-harm were similar for women (IR = 25.6 per 1000 person-years, 95% CI 20.7, 31.4) and men (IR = 25.5 per 1000 person-years, 95% CI 16.8, 37.1). There was no significant sex difference in the rate of ambulance attendance for self-harm (IRR = 1.1, 95% CI 0.52, 2.2; adjusted IRR = 0.67, 95% CI 0.3, 1.4).

## Discussion

We synthesised evidence on suicide, self-harm, and suicidal ideation among adults and youth after release from incarceration, and examined sex differences in these outcomes. Twenty-nine studies on suicide, two studies on self-harm, and one study on suicidal ideation met our inclusion criteria. Rates of suicide between women and men released from incarceration were similar, which contrasts with evidence from general population studies in which rates of suicide are typically higher among men than women [4]. We found that women released from incarceration have a risk of suicide that is almost 15 times greater than that of their general population counterparts. These findings have important implications for evidence-based suicide prevention efforts and transitional support for people released from incarceration, including services for both women and men that address their specific needs. Although evidence on self-harm and suicidal ideation among people released from incarceration is limited, the available findings indicate that high rates of these outcomes exist among this group. There is an urgent need for more high-quality research in these outcomes among people released from incarceration.

Although a high level of heterogeneity in a meta-analysis of observational studies is not unexpected [67], the amount of unexplained variance between studies in our findings means that they should be interpreted with some caution. The variance in our review may be due to a range of measured and unmeasured factors, such as underlying suicide risk across countries, the age of release from incarceration, access to various suicide methods across settings (e.g., firearm availability) and methodological factors (e.g., prospective or retrospective designs). Additionally, heterogeneity has been recognised as an issue in this area in a previous review of data linkage studies on mortality after release from incarceration [68]. This review recommended that to reduce avoidable heterogeneity, data linkage studies should ascertain deaths from a national death registry (rather than using state-based or coronial records) and exclude or adjust for subsequent periods of imprisonment [68]. Despite these recommendations having been made almost a decade ago, our study has found that these are ongoing methodological issues in this literature that potentially reduce study quality. Future research using linked data to examine suicide deaths after release from incarceration should consider such recommendations, to increase study quality, the utility of findings, and potential for evidence synthesis.

Our finding that people released from incarceration are more than seven times more likely than people in the general population to die by suicide is similar to findings from a previous meta-analysis on suicide after release from incarceration (RR = 6.8) [22]. Established predictors of suicide in the general population include, but are not limited to, unemployment [69], mental illness [70], homelessness [71], low socioeconomic status [72], and acute psychosocial stress [73]. These factors are common among people with a history of incarceration, and may be particularly pronounced in the weeks and months following release [13, 74, 75]. Further, a lack of continuity of care, including gaining or regaining access to mental health services in the community, is a common experience for people released from incarceration [76–78], and may contribute to their elevated suicide risk. A study from England and Wales found that increasing age over 25 years, release from a local prison, a history of alcohol misuse or self-harm, a psychiatric diagnosis, and requiring Community Mental Health Services (CMHS) follow-up after release from prison were all risk factors for suicide among people released from prison, while non-white ethnicity and a history of previous imprisonment were protective factors [79]. Similarly, a Swedish study found that a previous diagnosis of substance use disorder, previous suicide attempt and being born in Sweden (compared to being born abroad) were risk factors for suicide after release from incarceration [49]. Incarceration-level risk factors for suicide explored in previous research includes prison security level, with people released from high-security prisons experiencing an elevated risk of suicide compared to those released from low-security prisons [80]. Previous research on suicide risk during incarceration indicates that an interaction of social and incarceration-level factors (e.g., isolation) contribute to the risk of suicide during incarceration, rather than incarceration-level factors alone [1].

We found that suicide rates were similar between women and men released from incarceration. This is in contrast with the general population, where men have higher suicide rates than women [5]. Taken together, our findings may indicate that women released from incarceration are particularly vulnerable to suicide, because their rates of suicide are so high that they reach the same level as men released from incarceration. Consistent with this, we found that women released from incarceration are almost 15 times more likely than women in the general population to die by suicide. We found that, although both women and men released from incarceration are at increased risk of suicide relative to the same-sex general population, this elevation in risk is more than three times greater for women than for men (i.e., SMR of 14.9 and 4.6 for women and men, respectively).

Understanding the markedly elevated suicide risk among women released from incarceration compared to the general population requires examination of the potentially gender-specific risk factors for suicide to which women released from incarceration are exposed. Compared to both women in the general population and men released from incarceration, women released from incarceration experience substantially higher rates of homelessness [81, 82], substance use [19, 81], and mental illness [15, 83], which are established predictors of suicide [70, 71, 84]. Women released from incarceration may experience additional ‘gendered’ risk factors for suicide such as a history of childhood sexual abuse, trauma, and exposure to intimate partner violence [18]. These risk factors are more common among justice-involved women compared to both women in the general population [85–87] and justice-involved men [82, 88–90], and are also key drivers of female incarceration [88, 91]. These are also established risk factors for suicide [92–95]. Removal of one’s children is another risk factor for suicide that is more common among women with a history of incarceration compared to women in the general population [96–98]. There is evidence from the general population that exposure to more than one the aforementioned suicide risk factors compounds risk [99, 100]. Given that women released from incarceration typically experience a range of suicide risk factors [101, 102], the compounded and interacting effects of these exposures might explain, in part, their high suicide rates compared to women in the general population, and their similar rates to men released from incarceration. Targeted research, involving large and representative cohorts of women released from incarceration, are urgently required to explicate these pathways and inform prevention efforts tailored to women.

Our finding that suicide rates between women and men released from incarceration are similar does not necessarily mean that the same suicide prevention efforts will be effective for women and men in this population. The unique challenges women experience after release from incarceration [25, 101] must be considered as part of policy and planning. Existing transitional services are typically based on men’s needs and then applied to women [101] and, given the elevated risk of suicide among men and women released from incarceration, are evidently failing to adequately address both women’s and men’s suicide risk. A recent review of suicide prevention interventions among justice-involved people found limited evidence on interventions to address suicide risk, particularly for justice-involved women [103]. Another systematic review examining suicide prevention interventions among incarcerated people did not examine sex or gender differences at all [104]. More research is needed to inform gender-sensitive suicide prevention interventions to address both women’s and men’s unique needs, particularly among people in contact with the criminal justice system. Addressing the risk factors for why women enter incarceration, which overlap with risk factors for women’s suicide after release from incarceration (e.g., intimate partner violence, exposure to trauma, and removal of children) may be an effective way of reducing both women’s incarceration and suicide risk post-release.

Research on non-fatal suicidal outcomes after release from incarceration remains a critical gap in the literature. Our review identified just two studies on self-harm [64, 65] and one study on suicidal ideation [66] in people released from incarceration. The available findings indicate that people released from incarceration experience high rates of self-harm, with no difference by sex [64, 65]. This contrasts with the higher rates of self-harm and suicidal ideation among women in the general population compared to men. Although self-harm and suicidal ideation have been relatively well examined among incarcerated populations [3, 105], our study highlights the dearth of studies examining self-harm and suicidal ideation after incarceration. Further, although self-harm is often monitored in police custody or during incarceration [106], there does not appear to be monitoring of self-harm after release from incarceration, by health or justice agencies. This is despite evidence that the rates of these outcomes are an order of magnitude higher after incarceration than in custody [42]. Robust data on the incidence of self-harm and suicidal ideation after release from incarceration, including sex differences, are necessary to inform upstream transitional supports (e.g., addressing housing, supporting prosocial relationships, and early contact with mental health services), so that service providers can intervene as quickly as possible among both women and men at risk of suicide.

Our review is the most comprehensive to date to examine suicide, self-harm, and suicidal ideation after release from incarceration. We followed best-practice reporting guidelines [26] and excluded studies with small numbers of suicide deaths and short follow-up times; a conservative approach consistent with previous work [21]. The heterogeneity of estimates examined in our meta-analysis was high, and this was not accounted for by the factors examined in meta-regression. Due to the uneven distribution of covariates among studies, our meta-regression may lack sufficient statistical power to identify other sources of heterogeneity. All included studies reported on cohort studies, which is likely the strongest study design to examine the effect measures of interest in this population. However, there is considerable scope for methodological heterogeneity in cohort studies [68]. Our review was limited to studies published in peer-reviewed journals. However, there is some evidence that the inclusion of grey literature has a meaningful impact on meta-analyses in only a minority of reviews [107]. It is possible that including only English-language studies may have introduced some bias to our review, although there is evidence that excluding non-English studies does not have a meaningful impact on systematic review findings [108, 109]. All included studies were from high-income countries, which arguably precludes generalizing our findings to low- and middle-income countries (LMICs). High-quality evidence on suicide among people released from incarceration in LMICs is urgently needed.

Given the structure of the criminal justice system in most jurisdictions (i.e., incarcerating people by sex and not gender) and the scope of the available evidence, our review focused on sex differences and did not examine gender differences. There is some evidence that incarcerated transgender people have higher rates of suicide and self-harm compared to the general incarcerated population [110]. Future primary data collection studies on suicide, self-harm, and/or suicidal ideation in these settings should consider the experiences of transgender and gender diverse people.

People released from incarceration are more than seven times more likely than the general population to die by suicide. Women released from incarceration experience a particularly elevated risk of suicide compared to women in the general population. However, little is known about self-harm and suicidal ideation among this population, including differences between women and men. Our findings illustrate that suicide is not a ‘male problem’ only, particularly among people released from incarceration. Population-level suicide prevention policies must consider the needs of high-risk, marginalised groups, such as people released from incarceration, including the differences that exist between women and men.

These findings have important implications for the design and delivery of evidence-based transitional services for people released from incarceration that meet the needs of both women and men. Attention to the specific needs of women to reduce suicide risk is needed as part of these services. Along with robust, primary data collection about suicidal ideation and self-harm, more research is needed about people released from incarceration in LMICs, and about people who do not identify with their sex assigned at birth, to inform inclusive and effective suicide prevention policies and practices for these people who are marginalised.

## Supplementary Information

Below is the link to the electronic supplementary material.

Supplementary file1 (DOCX 5788 KB)

## Data Availability

Study data are available on request to the authors. The protocol for this review is available online via PROSPERO (CRD42020208885).
