# Supplementary material for: Sex differences in suicide, suicidal ideation, and self-harm after release from incarceration: a systematic review and meta-analysis
Source: Soc Psychiatry Psychiatr Epidemiol. 2022 Dec 3;58(3):355–71. doi: 10.1007/s00127-022-02390-z (PMC9971066; doi:10.1007/s00127-022-02390-z)
Supplement: Supplementary file 1 — Supplementary file1 (DOCX 5788 KB) [file 127_2022_2390_MOESM1_ESM.docx]

**Supplementary Tables**

[Table S1. Completed PRISMA checklist 2](#_Toc117161724)

[Table S2. Medline search strategy 5](#_Toc117161725)

[Table S3. Ascertainment and definitions of outcomes used by included studies 6](#_Toc117161726)

[Table S4. Summary of data extraction fields 8](#_Toc117161727)

[Table S5. Data sources used by included studies 9](#_Toc117161728)

[Table S6: Characteristics of participants from included studies 11](#_Toc117161729)

[Table S7. Findings from included studies reporting on sex as a risk factor for self-harm, suicidal ideation, and suicide after release from prison 19](#_Toc117161730)

[Table S8. Sensitivity analyses for pooled CMRs for non-sex-stratified samples, and for samples stratified by sex 20](#_Toc117161731)

[Table S9. Sensitivity analysis 1: Meta-regression for non-sex stratified samples CMR, including studies that reported the measures of interest but did not meet criteria for inclusion in primary analysis 22](#_Toc117161732)

[Table S10. Sensitivity analysis 2: Meta-regression for non-sex stratified samples CMR, restricting analysis to studies having a score above the median on the MASTER scale 24](#_Toc117161733)

[Table S11: Sensitivity analyses for pooled SMRs (non-sex stratified samples only) 26](#_Toc117161734)

**Supplementary Figures**

[Figure S1. PRISMA diagram of study selection. 13](#_Toc117162011)

[Figure S2. Forest plot for pooled suicide CMR per 100,000 person years for non-sex stratified samples 14](#_Toc117162012)

[Figure S3. Forest plot for pooled suicide CMR per 100,000 person years for women only 15](#_Toc117162013)

[Figure S4. Forest plot for pooled suicide CMR per 100,000 person years for men only 16](#_Toc117162014)

[Figure S5. Forest plot for pooled suicide SMR for women only 17](#_Toc117162015)

[Figure S6. Forest plot for pooled suicide SMR for men only 18](#_Toc117162016)

[Figure S7. Sensitivity Analysis 1: Non-sex stratified samples pooled CMR, including studies that reported the measures of interest but did not meet the criteria for inclusion in primary analysis 21](#_Toc117162017)

[Figure S8. Sensitivity Analysis 2: Non-sex stratified samples pooled CMR, restricting analysis to studies having a score above the median on the MASTER scale 23](#_Toc117162018)

[Figure S9. Sensitivity Analysis 3: Male-only pooled CMR, including studies that reported the measures of interest but did not meet the criteria for inclusion 25](#_Toc117162019)

[Figure S10. Sensitivity Analysis 4: Non-sex stratified samples pooled SMR, restricting analysis to studies having a score above the median on the MASTER scale 27](#_Toc117162020)

Table S1. Completed PRISMA checklist

| **Section and Topic** | **Item #** | **Checklist item** | **Location where item is reported (page)** |
| --- | --- | --- | --- |
| **TITLE** | | |  |
| Title | 1 | Identify the report as a systematic review. | 1 |
| **ABSTRACT** | | |  |
| Abstract | 2 | See the PRISMA 2020 for Abstracts checklist. | 1 |
| **INTRODUCTION** | | |  |
| Rationale | 3 | Describe the rationale for the review in the context of existing knowledge. | 3 |
| Objectives | 4 | Provide an explicit statement of the objective(s) or question(s) the review addresses. | 3 |
| **METHODS** | | |  |
| Eligibility criteria | 5 | Specify the inclusion and exclusion criteria for the review and how studies were grouped for the syntheses. | 3-4, Table S3 |
| Information sources | 6 | Specify all databases, registers, websites, organisations, reference lists and other sources searched or consulted to identify studies. Specify the date when each source was last searched or consulted. | 3 |
| Search strategy | 7 | Present the full search strategies for all databases, registers and websites, including any filters and limits used. | 3, Table S2 |
| Selection process | 8 | Specify the methods used to decide whether a study met the inclusion criteria of the review, including how many reviewers screened each record and each report retrieved, whether they worked independently, and if applicable, details of automation tools used in the process. | 4 |
| Data collection process | 9 | Specify the methods used to collect data from reports, including how many reviewers collected data from each report, whether they worked independently, any processes for obtaining or confirming data from study investigators, and if applicable, details of automation tools used in the process. | 4,9 |
| Data items | 10a | List and define all outcomes for which data were sought. Specify whether all results that were compatible with each outcome domain in each study were sought (e.g. for all measures, time points, analyses), and if not, the methods used to decide which results to collect. | 3-4, Table S3 |
|  | 10b | List and define all other variables for which data were sought (e.g. participant and intervention characteristics, funding sources). Describe any assumptions made about any missing or unclear information. | 4, Table S4-S5 |
| Study risk of bias assessment | 11 | Specify the methods used to assess risk of bias in the included studies, including details of the tool(s) used, how many reviewers assessed each study and whether they worked independently, and if applicable, details of automation tools used in the process. | 4 |
| Effect measures | 12 | Specify for each outcome the effect measure(s) (e.g. risk ratio, mean difference) used in the synthesis or presentation of results. | 4 |
| Synthesis methods | 13a | Describe the processes used to decide which studies were eligible for each synthesis (e.g. tabulating the study intervention characteristics and comparing against the planned groups for each synthesis (item #5)). | 4 |
|  | 13b | Describe any methods required to prepare the data for presentation or synthesis, such as handling of missing summary statistics, or data conversions. | 4 |
|  | 13c | Describe any methods used to tabulate or visually display results of individual studies and syntheses. | 4 |
|  | 13d | Describe any methods used to synthesize results and provide a rationale for the choice(s). If meta-analysis was performed, describe the model(s), method(s) to identify the presence and extent of statistical heterogeneity, and software package(s) used. | 4 |
|  | 13e | Describe any methods used to explore possible causes of heterogeneity among study results (e.g. subgroup analysis, meta-regression). | 4 |
|  | 13f | Describe any sensitivity analyses conducted to assess robustness of the synthesized results. | 4-5 |
| Reporting bias assessment | 14 | Describe any methods used to assess risk of bias due to missing results in a synthesis (arising from reporting biases). | N/A |
| Certainty assessment | 15 | Describe any methods used to assess certainty (or confidence) in the body of evidence for an outcome. | N/A |
| **RESULTS** | | |  |
| Study selection | 16a | Describe the results of the search and selection process, from the number of records identified in the search to the number of studies included in the review, ideally using a flow diagram. | 5, Fig S1 |
|  | 16b | Cite studies that might appear to meet the inclusion criteria, but which were excluded, and explain why they were excluded. | 5, Fig S1 |
| Study characteristics | 17 | Cite each included study and present its characteristics. | 5, Table 1 |
| Risk of bias in studies | 18 | Present assessments of risk of bias for each included study. | Table 1 |
| Results of individual studies | 19 | For all outcomes, present, for each study: (a) summary statistics for each group (where appropriate) and (b) an effect estimate and its precision (e.g. confidence/credible interval), ideally using structured tables or plots. | Table 1-2, Table S6 |
| Results of syntheses | 20a | For each synthesis, briefly summarise the characteristics and risk of bias among contributing studies. | Table 1 |
|  | 20b | Present results of all statistical syntheses conducted. If meta-analysis was done, present for each the summary estimate and its precision (e.g. confidence/credible interval) and measures of statistical heterogeneity. If comparing groups, describe the direction of the effect. | 5-6, Tables 1-3 and Figures 1-3, Tables S6-11, Figures S2-10 |
|  | 20c | Present results of all investigations of possible causes of heterogeneity among study results. | 5-6, Table 3 |
|  | 20d | Present results of all sensitivity analyses conducted to assess the robustness of the synthesized results. | 6, Tables S8–11, Figures S7–S10 |
| Reporting biases | 21 | Present assessments of risk of bias due to missing results (arising from reporting biases) for each synthesis assessed. | N/A |
| Certainty of evidence | 22 | Present assessments of certainty (or confidence) in the body of evidence for each outcome assessed. | N/A |
| **DISCUSSION** | | |  |
| Discussion | 23a | Provide a general interpretation of the results in the context of other evidence. | 6-9 |
|  | 23b | Discuss any limitations of the evidence included in the review. | 8 |
|  | 23c | Discuss any limitations of the review processes used. | 8 |
|  | 23d | Discuss implications of the results for practice, policy, and future research. | 8-9 |
| **OTHER INFORMATION** | | |  |
| Registration and protocol | 24a | Provide registration information for the review, including register name and registration number, or state that the review was not registered. | 3 |
|  | 24b | Indicate where the review protocol can be accessed, or state that a protocol was not prepared. | 3 |
|  | 24c | Describe and explain any amendments to information provided at registration or in the protocol. | 3, 4-5 |
| Support | 25 | Describe sources of financial or non-financial support for the review, and the role of the funders or sponsors in the review. | 2 |
| Competing interests | 26 | Declare any competing interests of review authors. | 2 |
| Availability of data, code and other materials | 27 | Report which of the following are publicly available and where they can be found: template data collection forms; data extracted from included studies; data used for all analyses; analytic code; any other materials used in the review. | 9 |

Table S2. Medline search strategy

| **Search number** | **Search Term** |
| --- | --- |
| #1 | *Criminals/ or *Prisoners/ or *criminal justice/ |
| #2 | (parole* or prison* or jail* or justice-involved or custodial or correctional or incarcerat* or detention or detain* or convict* or ex-offend* or exoffend* or ex-prison* or exprison* or post-prison* or ex-con* or excon* or ex-felon* or former-inmate* or ex-inmate* or parole* or leaving-prison? or leaving-jail? or (release* adj2 prison?) or (release* adj2 jail?) or former-prisoner*).tw,kf,hw. |
| #3 | *self-injurious behavior/ or *self mutilation/ or *suicide/ or *suicidal ideation/ or *suicide, attempted/ or *suicide, completed/ |
| #4 | (((self or themsel*) adj2 (injur* or mutilat* or harm*3 or wound* or violen* or strangl* or strangu* or poison* or overdos* or cut or cutting or hang*3)) or self-inflict* or NSSI or DSH or parasuicid* or para-suicid* or ((deliberate or intentional) adj6 (poison* or overdos*))).tw,kf,hw. |
| #5 | (suicid* or (suicid* adj2 attempt*) or (suicid* adj2 thought*) or (suicid* adj2 behav*) or (suicid* adj2 idea*)).tw,kf,hw. |
| #6 | (1 or 2) and (3 or 4 or 5) |
| #7 | exp cohort studies/ |
| #8 | (longterm or long-term or repeat* or serial or longitudinal* or follow-up or followup or cohort* or retrospective* or prospective*).tw,kf,hw. |
| #9 | case-control studies/ |
| #10 | Epidemiologic Methods/ |
| #11 | Cross-Sectional Studies/ |
| #12 | ((control* adj clinical adj trial*) or (case* adj control*) or cross-section*).tw,kf,hw. |
| #13 | 7 or 8 or 9 or 10 or 11 or 12 |
| #14 | 6 and 13 |
| #15 | limit 14 to (case reports or comment or editorial or letter) |
| #16 | 14 not 15 |
| #17 | limit 16 to (english language and yr="1970 -Current") |

Table S3. Ascertainment and definitions of outcomes used by included studies

| **First author, year** | **Outcome** | **Ascertainment of outcome** | **Definition of outcome (e.g., specific ICD codes)** |
| --- | --- | --- | --- |
| Barnert, 2019 [1] | Suicidal ideation | National Longitudinal study of Adolescent to Adult Health survey data (self-report) | Single-item, dichotomous survey question asking participants if they had ‘seriously considered suicide’ in the previous 12 months. |
| Borschmann, 2017a [2] | Self-harm | Emergency Department (ED) records | Cutting and/or burning (excluding self-piercing and tattooing), self-poisoning (i.e., ingesting drugs or substances that are typically not used recreationally (e.g., sedatives) for the purpose of overdosing), self-battering (i.e., punching or striking any part of the body with an object or another part of the body), risk-taking (i.e., any intentional, non-recreational risks that served no legitimate social purpose and which resulted in actual harm to the participant) and other (attempted self-drowning, hanging, intentional electrocution, suffocation or overtly specified suicide attempts). |
| Borschmann, 2017b [3] | Self-harm | Queensland Ambulance Service data | An act with a non-fatal outcome in which an individual deliberately initiates behaviour (such as self-cutting) or ingesting an illicit drug or non-ingestible substance or object, with the intention of causing harm to themselves. Includes poisoning with any licit substance. |
| Binswanger, 2007 [4] | Suicide | National Death Index records | ICD-10 coding (codes not specified, however stated that definition included accidental poisoning and exposure to noxious substances as overdose). |
| Binswanger, 2013 [5] | Suicide | National Death Index records | ICD-10 coding (codes not specified) |
| Bird, 2003 [6] | Suicide | General Register Office for Scotland death records | ICD-9 coding (E950-E959) |
| Brinkley-Rubinstein, 2019 [7] | Suicide | North Carolina death records | ICD-10 coding (X60-X84, Y87.0). |
| Bukten, 2019 [8] | Suicide | Norwegian Death Registry | ICD-10 coding ((X65–X84, Y87.0). |
| Bukten, 2021 [9] | Suicide | Norwegian Cause of Death Register | ICD-10 coding (X60-84) |
| Chang, 2015 [10] | Suicide | Swedish Cause of Death Register | ICD-10 coding for suicide, including undetermined deaths (Y10–Y34). |
| Coffey, 2003 [11] | Suicide | Australian National Death Index records | ICD-9 and -10 coding (codes not specified) |
| Coffey, 2004 [12] | Suicide | Australian National Death Index records | ICD-9 and -10 coding (codes not specified) |
| Dirkzwager, 2012 [13] | Suicide | Netherlands National Death Index | ICD-10 coding (codes not specified) |
| Farrell, 2007 [14] | Suicide | National Health Service Central Register data | ICD-9 and -10 coding (codes not specified) |
| Graham 2003 [15] | Suicide | Victorian Coronial data | Not described |
| Haglund, 2014 [16] | Suicide | Swedish Cause of Death Register | ICD-8, -9 and -10 coding for certain suicide and unnatural death with undetermined intent (ICD-8 and ICD-9: E950-9, E980-9, ICD-10:X60-84, Y10-34) |
| Harding-Pink, 1990 [17] | Suicide | Autopsy records, notes from the medical service of the prison from which participants were discharged | Not described |
| Jones, 2017 [18] | Suicide | North Carolina death records | ICD-10 coding (codes not specified). |
| Kariminia, 2007a, b [19,20] | Suicide | Australian National Death Index records | ICD-8, -9 and -10 coding (ICD-9 E950.0–E950.5; ICD-10 X60–X64) |
| Kouyoumdjian, 2016 [21] | Suicide | Registered Persons Database records | ICD-9 coding for suicide and self-inflicted injury (E950-E959, X60-X84) |
| Lim, 2012 [22] | Suicide | NYC Department of Health and Mental Hygiene Vital Statistics Registry | ICD-10 coding (X60–X84 and X87.0) |
| Pratt, 2006 [23] | Suicide | Database of the National Confidential Inquiry into Suicide and Homicide by People with Mental Illness for England and Wales and Home Office Register | The authors classified suicide and open verdicts as suicides. |
| Rosen, 2008 [24] | Suicide | North Carolina Center for  Health Statistics records | ICD-9 and -10 coding (codes not specified) |
| Rosen, 2020 [25] | Suicide | North Carolina Center for  Health Statistics records | ICD-10 coding (codes not specified) |
| Sailas, 2005 [26] | Suicide | Statistics Finland’s Cause of Death Register | ICD-8, -9 and -10 coding (codes not specified) |
| Spittal, 2014 [27] | Suicide | Australian National Death Index records | ICD-9 and -10 coding (ICD-9: E950-E959; ICD-10 X60-X84). |
| Stewart, 2004 [28] | Suicide | Registrar-General’s record of deaths | ICD-9 and -10 coding (ICD9: E950-E959, ICD10: X60-X84) |
| van Dooren, 2013 [29] | Suicide | Australian National Death Index records | ICD-9 and -10 coding (ICD-9: E950-E959; ICD-10 X60-X84). |

Table S4. Summary of data extraction fields

| **Domain** | **Data extraction field** |
| --- | --- |
| **Study details** | Authors |
|  | Year of publication |
|  | Title of paper |
|  | Journal name |
| **Study design** | Geographical location of study |
|  | Year(s) of study |
|  | Prospective/retrospective design |
|  | Sample selection (e.g., single sex samples) |
|  | Median and total length of follow-up |
|  | Whether the time and events in subsequent periods of incarceration during follow-up were removed from analysis (i.e., interval truncation) |
|  | Type of incarceration (e.g., youth justice detention, prison, jail) |
| **Cohort characteristics** | Cohort size (n) |
|  | Number and proportion of males and females in the cohort |
|  | Total person-years at risk of death, overall and stratified by sex |
|  | Age of people in the cohort at baseline, overall and stratified by sex |
|  | Number and proportion of people from a marginalised ethnic group in the cohort, overall and stratified by sex  Record source for incarceration (e.g., correctional records) |
| **Outcomes** | Record source for outcome (e.g., death records) |
|  | Definition of suicide, self-harm or suicidal ideation |
|  | Number of suicides in the cohort, overall and stratified by sex |
|  | Number of people experiencing self-harm in the cohort, overall and stratified by sex |
|  | Number of self-harm events in the cohort, overall and stratified by sex |
|  | Number of people experiencing suicidal ideation in the cohort, overall and stratified by sex |
|  | Suicide crude mortality rate, overall and stratified by sex |
|  | Suicide standardised mortality ratio, overall and stratified by sex |
|  | Reference populations |
|  | Incidence of self-harm, overall and stratified by sex |
|  | Incidence of suicidal ideation, overall and stratified by sex |
|  | Findings on sex as a risk factor for suicide (any measure of association) |
|  | Findings on sex as a risk factor for self-harm (any measure of association) |
|  | Findings on sex as a risk factor for suicidal ideation (any measure of association) |

Table S5. Data sources used by included studies

| **First author, year** | **Correctional data source** | **Outcome data source for people released from prison** | **Outcome data source for matched reference population** |
| --- | --- | --- | --- |
| Barnert, 2019 [1] | National Longitudinal study of Adolescent to Adult Health survey data (self-report) | National Longitudinal study of Adolescent to Adult Health survey data (self-report) | NA |
| Binswanger, 2007 [4] | Washington State Department of Corrections | National Death Index records | Centers for Disease Control and Prevention Wide-ranging Online Data for Epidemiologic Research (CDC WONDER) |
| Binswanger, 2013 [5] | Washington State Department of Corrections | National Death Index records | Centers for Disease Control and Prevention Wide-ranging Online Data for Epidemiologic Research (CDC WONDER) |
| Bird, 2003 [6] | Scottish Prisoner Information Network | General Register Office for Scotland | General Register Office for Scotland |
| Borschmann, 2017a [2] | Queensland Correctional Service records | Linked state-level Emergency Department (ED) records | NA |
| Borschmann, 2017b [3] | Queensland Correctional Service records | Queensland Ambulance Service (QAS) data | NA |
| Brinkley-Rubinstein, 2019 [7] | North Carolina Department of Public Safety (NCDPS) | North Carolina death records | NA |
| Bukten, 2017 [8] | Norwegian Prison Registry | Norwegian Death Registry | NA |
| Bukten, 2021 [9] | Norwegian Prison Registry data from Norwegian Prison Release study (nPRIS) | Norwegian Cause of Death Register | NA |
| Chang, 2015 [10] | Swedish National Crime Register | Swedish Cause of Death Register | NA |
| Coffey, 2003 [11] | Victorian Department of Human Services and Victorian Department of Justice | Australian National Death Index records | Australian Bureau of Statistics |
| Coffey, 2004 [12] | Victorian Department of Human Services and Victorian Department of Justice | Australian National Death Index records and Victorian coroner | NA |
| Dirkzwager, 2012 [13] | Netherlands Public Prosecutor’s Office | Netherlands National Death Index | Netherlands National Death Index |
| Farrell, 2007 [14] | Population databases operated by the Prison Service and Home Office | National Health Service Central Register | NA |
| Graham, 2003 [15] | Victorian Prisoner Information Management System | Victorian Coronial database | Australian Bureau of Statistics |
| Haglund, 2014 [16] | Swedish Prison Register | Swedish Cause of Death Register | Swedish Cause of Death Register |
| Harding-Pink, 1990 [17] | Prison administration records | Autopsy records, notes from the medical service of the prison from which participants were discharged | NA |
| Jones, 2017 [18] | North Carolina Department of Public Safety, Office of Rehabilitative Programs and Services | North Carolina death records | NA |
| Kariminia, 2007a, b [19,20] | New South Wales (NSW) Department of Corrective Services Offender Integrated Management System (OIMS) | Australian National Death Index records | Australian National Death Index (NSW only) |
| Kouyoumdjian, 2016 [21] | Ontario Ministry of Community Safety and Correctional Services database | Ontario Registrar General Death database | Statistics Canada |
| Lim, 2012 [22] | NYC Department of Health and Mental Hygiene Vital Statistics registry | NYC Department of Health and Mental Hygiene Vital Statistics registry | US Census data |
| Pratt, 2006 [23] | Inmate Information System, Home Office, England | Inmate Information System, Home Office, England | Inmate Information System, Home Office, England |
| Rosen, 2008 [24] | North Carolina Department of Corrections electronic imprisonment records | North Carolina Center for Health Statistics records | North Carolina population Census data |
| Rosen, 2020 [25] | North Carolina prison system | North Carolina Center for Health Statistics records | NA |
| Sailas, 2005 [26] | Prison Court Register | Statistics Finland’s Cause of Death Register | NA |
| Spittal, 2014 [27] | Queensland Correctional Service records | Australian National Death Index records | Australian National Death Index records |
| Stewart, 2004 [28] | Ministry of Justice (Western Australia) | Registrar-General’s record of deaths (Western Australia) | Registrar-General’s record of deaths (Western Australia) |
| van Dooren, 2013 [29] | Queensland Correctional Service records | Australian National Death Index records | Australian Bureau of Statistics |

Table S6: Characteristics of participants from included studies

| **Author, year** | **Participants n (%)** | | | **Age at baseline (years)** | | | **Ethnic and racial minorities n (%)** | | | |
| --- | --- | --- | --- | --- | --- | --- | --- | --- | --- | --- |
|  | **All** | **Men** | **Women** | **All** | **Men** | **Women** | **Reported ethnic or racial minorit(ies)** | **All** | **Men** | **Women** |
| Barnert, 2019 [1] | 1727 | 1344 (77.8) | 383 (22.2) | Range:  7-24 | NR | NR | Black or Hispanic people | 577 (33.4) | NR | NR |
| Binswanger, 2007 [4] | 30,237 | 26270 (87) | 3697 (13) | Mean: 33.4±9.8 (Range: 18-84) | NR | NR | Non-white people (includes Black and non-Hispanic, Hispanic only, Native American, Alaska Native, non-Hispanic Asian or Pacific Islander) | 11380 (38) | NR | NR |
| Binswanger, 2013 [5] | 76,208 | 63979 (84.0) | 12229 (16.0) | Range: 18-84 | NR | NR | African American people, Hispanic people, Asian people and others | 27002 (36.4) | NR | NR |
| Bird, 2003 [6] | 19,486 | 19,486  (100.0) | NA | Range: 15-35 | 15-35 | NA | NR | NR | NR | NA |
| Borschmann, 2017a [2] | 1307 | 1030 (78.8) | 277 (21.2) | Range: 18-40+ | NR | NR | Indigenous people | 331 (25.3) | NR | NR |
| Borschmann, 2017b [3] | 1309 | 976 | 260 | Range: 18-40+ | NR | NR | Indigenous people | 307 (24.8) | NR | NR |
| Brinkley-Rubinstein, 2019 [7] | 229274 | 197656 (86.2) | 31618 (13.8) | Median: 32 (IQR: 26-42) | NR | NR | Non-white people | 136,597 | NR | NR |
| Bukten, 2017 [8] | 92,663 | (Range: 90.0-92.0) | (Range: 8.0-10.0) | NR | NR | NR | NR | NR | NR | NR |
| Bukten, 2021 [9] | NR | NR | NR | Median: 31 (IQR: 23-41) | NR | NR | NR | NR | NR | NR |
| Chang, 2015 [10] | 47326 | 43840 | 3486 | NR | NR | NR | People who immigrated | 14516 | 13710 (31) | 806 (23) |
| Coffey, 2003 [11] | 2,849 | 2621 (92.0) | 228 (8.0) | NR | Median: 17.9 | Median: 18.4 | NR | NR | NR | NR |
| Coffey, 2004 [12] | 2,849 | 2625 (92.0) | 228 (8.0) | NR | Range: 10-20 | Range: 10-20 | Indigenous Australian people | 1115** | NR | NR |
| Dirkzwager, 2012 [13] | 597 | 578 (96.8) | 19 (3.2) | Range: 12-40+ | NR | NR | Non-Dutch people | 102 (17.1) | NR | NR |
| Farrell, 2007 [14] | 48771 | 36513 (74.9) | 12258 (25.1) | 15+ years | NR | NR | NR | NR | NR | NR |
| Graham, 2003 [15] | 25,469 | 22,978 (90.2) | 2490 (9.8) | NR | NR | NR | NR | NR | NR | NR |
| Haglund, [16] | 38995 | 36146 (92.7) | 2849 (7.3) | Mean: 37.8 (SD: 12.1, range:  18-84) | Range: 17-65+ | Range: 17-65+ | Born overseas | 10831  (27.8) | 10226 (28.3) | 605 (27.8) |
| Harding-Pink, 1990 [17] | NR | NR | NR | NR | NR | NR | NR | NR | NR | NR |
| Jones, 2017 [18] | 41495 | 37053 (89.3) | 4442 (10.7) | Range: 20-60+ | NR | NR | Black people | 25467 (61.4) | NR | NR |
| Kariminia, 2007a, b [19,20] | 85203 | 76383 (89.6) | 8820 (10.4) | NR | Median: 27.2 (Range: 18-86) | Median: 27.3 (Range: 18-83) | Aboriginal Australian | 9353  (7.75) | 7980 (10.4) | 1373 (15.6) |
| Kouyoumdjian, 2016 [21] | 48166 | 43419 (90.1) | 4747 (9.9) | NR | Median: 32 | Median: 33 | Black or Aboriginal people | Black: 6330 (12.4) Aboriginal: 3465 (7.2) | Black: 5374 (12.4) Aboriginal: 3005 (6.9) | Black:  596 (12.6) Aboriginal: 460 (9.7) |
| Lim, 2012 [22] | 155,272 | 137161 (88) | 18,111 (12) | Range: 16-89 | NR | NR | Non-Hispanic Black people, Hispanic people, Asian people, | 141789 (91) | NR | NR |
| Pratt, 2006 [23] | NR | NR | NR | Range: 18-50+ | NR | NR | NR | NR | NR | NR |
| Rosen, 2008 [24] | 168,001 | 168,001 (100.0) | NA | Median: 32  (IQR:25–40, range: 20-69). | Median: 32  (IQR:25–40, range: 20-69). | NA | Black people | 92401 (55.0) | 92401 (55.0) | NA |
| Rosen, 2020 [25] | 111479 | 96367 (86.4) | 15112 (13.6) | Range: 18-88 | NR | NR | Black people,  Hispanic people,  Non-Hispanic people /other/unknown | 64658 (58.0) | NR | NR |
| Sailas, 2005 [26] | 3,832 | 3743 (97.7) | 89 (2.3) | Range: 15-21 | NR | NR | NR | NR | NR | NR |
| Spittal, 2014 [27] | 41970 | 36994 (88.1) | 4976 (12.9) | Range: 17-40+ | NR | NR | Indigenous Australian people | 8015 (19.1) | NR | NR |
| Stewart, 2004 [28] | 9,381 | 8199 (87.4) | 1182 (13.6) | Mean: 31, median: 29,  mode: 21 (range: 16-82) | NR | NR | Aboriginal Australian people | 2526 (26.9) | 1972 | 554 |
| van Dooren, 2013 [29] | 42,015 | 37039 (88.2) | 4976 (11.8) | NR | NR | NR | Indigenous Australian people | 8021 (19.1) | 6808 (84.9) | 1213 (15.1) |
| NR = Not reported. | | | | | | | | | | |

Reports not retrieved

(n = 1)

Records screened

(n = 1,711)

Records excluded**

(n = 1,556)

Records removed before screening:

Duplicate records removed

(n = 1,573)

Records identified from:

Databases (n = 3,248)

Other sources (n = 36)

Reports sought for retrieval

(n = 155)

**Identification**

Full-text articles excluded (n=127)
*No outcome of interest (n=35)*

*Insufficient data (n=24)*

*Selected sample (n=21)*

*Sample not released from prison (n=17)*

*Sample not disaggregated by incarceration status (n=15)*

*Sample does not have incarceration history (n=7)*

*Not a journal article (n=6)*

*Composite measure of suicide that included other outcomes (e.g., depression) (n=1)*

*Literature review (n=1)*

Studies included in review

(n = 29)

Reports assessed for eligibility

(n = 154)

Studies included in quality assessment

(n = 29)

**Screening**

Records identified through citation search

(n = 2)

**Included**

Figure S1. PRISMA diagram of study selection.

**Caption:** This is a flowchart diagram presenting the results at each stage of the review process. A total of 3248 records were identified through database searching, and an additional 36 records through other sources. In total, 3284 records were checked for duplicates, and 1573 duplicates were removed. A total of 1711 records were title and abstract screened, and 1556 of these records were excluded. This left 155 full-text articles assessed for eligibility, of which 128 were excluded. The 128 articles were excluded for the following reasons: No outcome of interest (n=35); Insufficient data (n=24); Selected sample (n=21); Sample not released from prison (n=17); Sample not disaggregated by incarceration status (n=15); Sample does not have incarceration history (n=7); Not a journal article (n=6); Composite measure of suicide that included other outcomes (e.g., depression) (n=1); Literature review (n=1); and Full-text not available (n=1). Two additional studies were identified through citation searching and added to the review. Therefore, 29 studies were assessed for quality and included in the review.


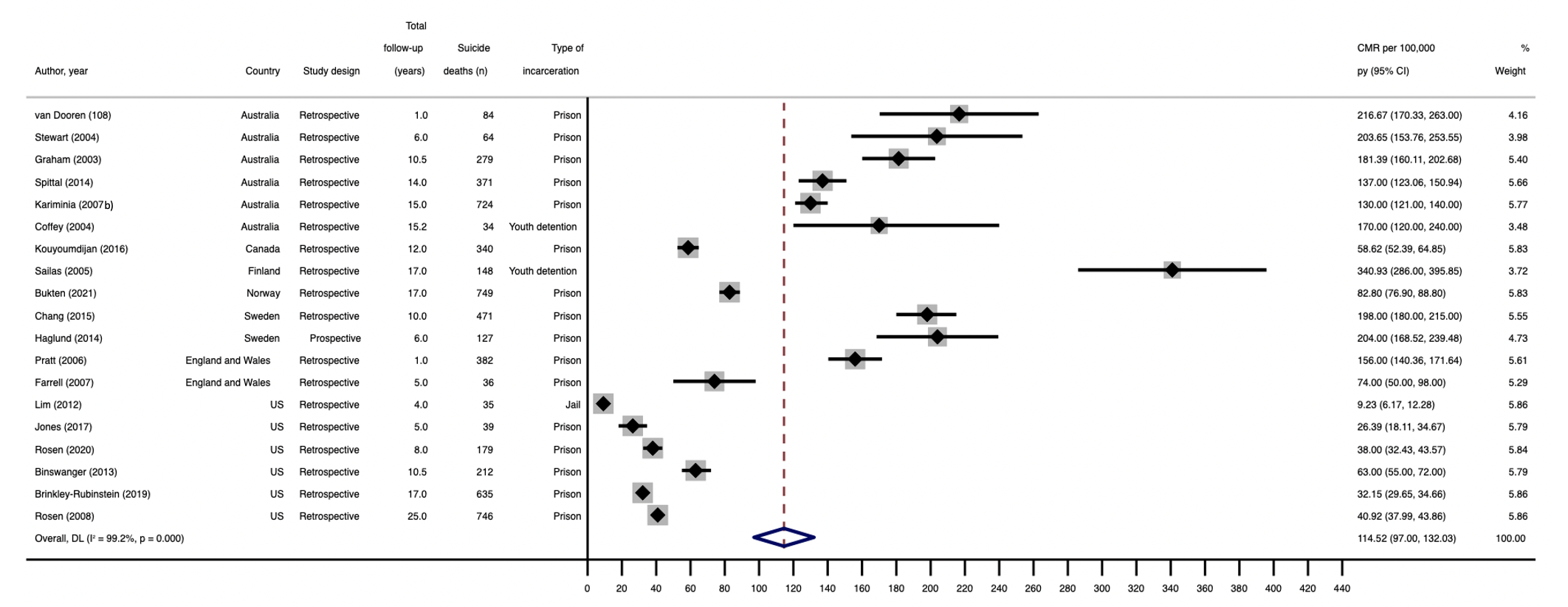


Figure S2. Forest plot for pooled suicide CMR per 100,000 person years for non-sex stratified samples


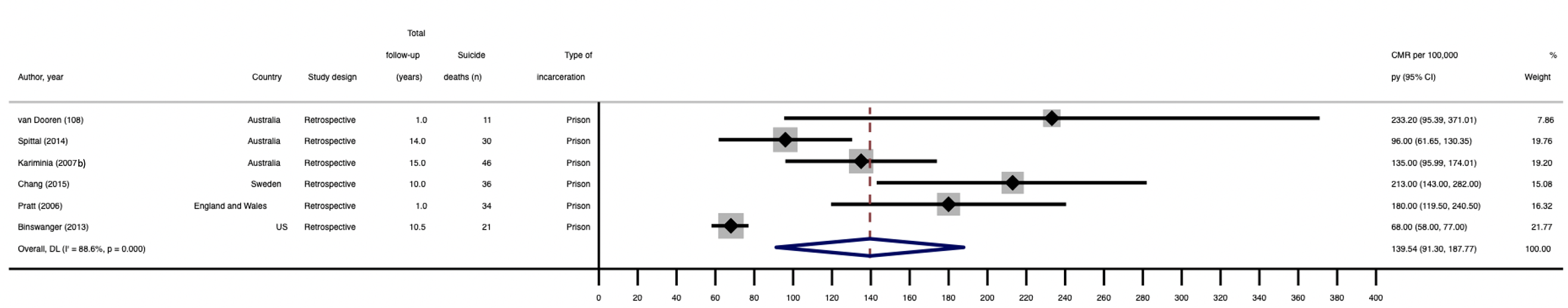


Figure S3. Forest plot for pooled suicide CMR per 100,000 person years for women only


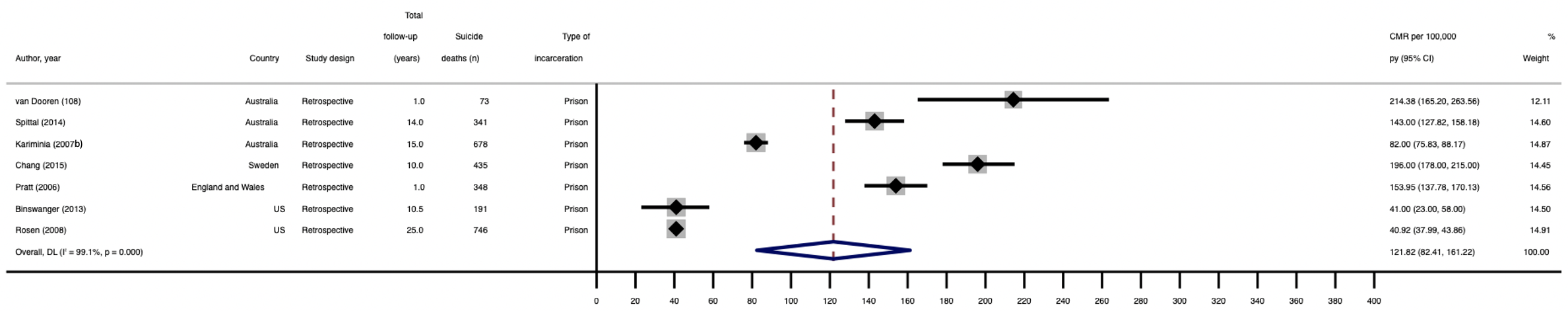


Figure S4. Forest plot for pooled suicide CMR per 100,000 person years for men only


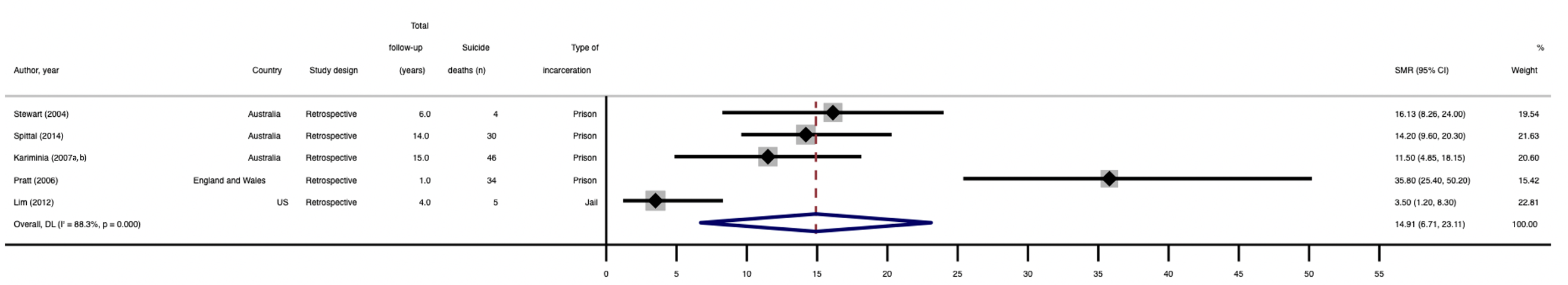


Figure S5. Forest plot for pooled suicide SMR for women only


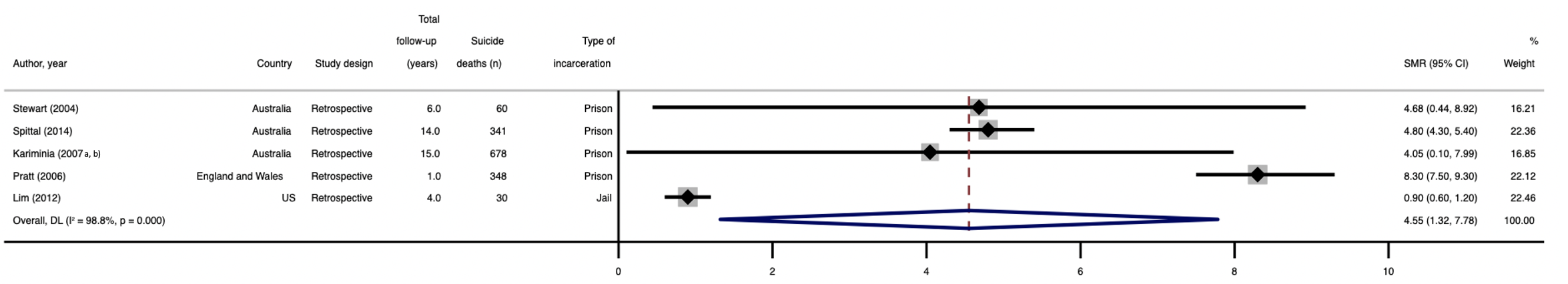


Figure S6. Forest plot for pooled suicide SMR for men only

Table S7. Findings from included studies reporting on sex as a risk factor for self-harm, suicidal ideation, and suicide after release from prison

| **First author, year** | **Outcome** | **Effect measure** | **Reference group** | **Finding (95%CI)** | **p-value** |
| --- | --- | --- | --- | --- | --- |
| Bukten, 2021 [9] | Suicide | Hazard Ratio | Women vs. men | 1.19 (0.89-1.58) | 0.239 |
| Haglund, 2014 [16] | Suicide | Adjusted hazard ratio | Men vs. women | 1.1 (0.6-1.9) | NR |
| Lim, 2012 [22] | Suicide | Risk ratio | Women vs. men | 0.7 (0.4-1.2) | NR |
| Pratt, 2006 [23] | Suicide | Age-adjusted rate ratio | Men vs. women | 1.1 (0.8-1.6) | 0.469 |
| Spittal, 2014 [27] | Suicide | Adjusted hazard ratio | Women vs. men | 1.4 (1.0-2.1) | 0.059 |
| van Dooren, 2013 [29] | Suicide | Hazard ratio | Women vs. men | 1.3 (0.9-1.9) | 0.11 |
| NR= Not reported. | | | | | |

Table S8. Sensitivity analyses for pooled CMRs for non-sex-stratified samples, and for samples stratified by sex

|  | **Number of studies** | **Suicide CMR (95%CI)**  **per 100,000 person years** | **I^2^** |
| --- | --- | --- | --- |
| Non-sex stratified samples CMR |  |  |  |
| Primary analysis | 19 | 114.5 (97.0-132.0) | 99.2 |
| Sensitivity analysis 1: Including studies that reported the measures of interest but did not meet the criteria for inclusion in primary analysis | 21 | 114.0 (96.8-131.1) | 99.2 |
| Sensitivity analysis 2: Restricting analysis to studies having a score above the median on the MASTER scale | 14 | 114.1 (93.7-134.5) | 99.3 |
| Male-only CMR |  |  |  |
| Primary analysis | 7 | 121.8 (82.4-161.2) | 99.1 |
| Sensitivity analysis 3: Analysis including studies that reported the measures of interest but did not meet the criteria for inclusion in primary analysis* | 8 | 127.6 (88.8-166.3) | 99.0 |

Note: CMR=crude mortality rate. There were no female-only papers that reported measures of interest but did not meet the criteria for inclusion. There were no female-only papers or male-only papers that included papers with a higher risk of bias. *As there were fewer than 10 included studies, meta-regression was not possible.


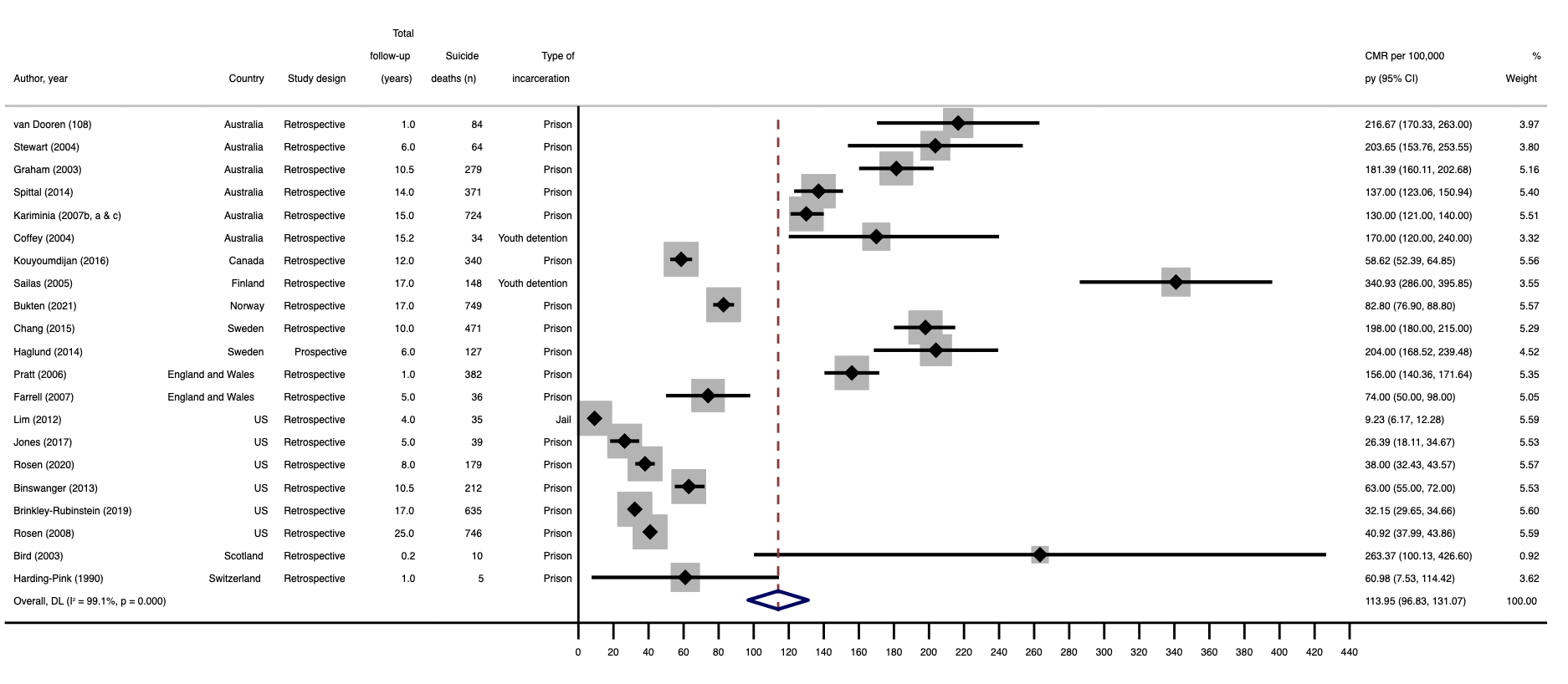


Figure S7. Sensitivity Analysis 1: Non-sex stratified samples pooled CMR, including studies that reported the measures of interest but did not meet the criteria for inclusion in primary analysis

Table S9. Sensitivity analysis 1: Meta-regression for non-sex stratified samples CMR, including studies that reported the measures of interest but did not meet criteria for inclusion in primary analysis

| **Factor** | **Number of studies** | **CMR (95% CI) per 100,000 person years** | **p-value** | **I^2^** |
| --- | --- | --- | --- | --- |
| **Type of incarceration facility** |  |  |  |  |
| Jail | 1 | 9.23 (0.00, 162.87) | 0.0323 | 98.89% |
| Prison | 18 | 114.50 (77.16, 151.84) |  |  |
| Youth detention | 2 | 256.54 (139.16, 373.93) |  |  |
|  |  |  |  |  |
| **Study design** |  |  |  |  |
| Prospective | 1 | 204.00 (21.40, 386.60) | 0.352 | 99.12% |
| Retrospective | 20 | 118.66 (77.66, 159.66) |  |  |
|  |  |  |  |  |
| **Interval censoring** |  |  |  |  |
| No | 9 | 139.47 (76.28, 202.65) | 0.483 | 99.14% |
| Yes | 12 | 111.38 (58.75, 164.00) |  |  |
|  |  |  |  |  |
| **Total length of follow-up (years)^a^** |  |  |  |  |
| ≤10 years | 11 | 124.87 (67.53, 182.22) | 0.922 | 99.08% |
| >10 years | 10 | 120.96 (62.36, 179.56) |  |  |
|  |  |  |  |  |
| **Country** |  |  |  |  |
| Australia | 6 | 166.45 (137.75, 195.14) | <0.001 | 96.74% |
| Canada | 1 | 58.62 (0.00, 118.61) |  |  |
| Finland | 1 | 340.93 (255.37, 426.49) |  |  |
| Norway | 1 | 82.80 (22.85, 142.75) |  |  |
| Sweden | 2 | 200.61 (153.44, 247.78) |  |  |
| England and Wales | 2 | 117.09 (72.07, 162.10) |  |  |
| US | 6 | 34.87 (10.41, 59.34) |  |  |
| Scotland | 1 | 263.37 (71.40, 455.33) |  |  |
| Switzerland | 1 | 60.98 (0.00, 145.36) |  |  |
|  |  |  |  |  |
| **Male only samples** |  |  |  |  |
| No | 19 | 123.44 (80.82, 166.07) | 0.927 | 99.16% |
| Yes | 2 | 116.57 (0.00, 265.27) |  |  |

| Note: CMR = crude mortality rate; 95%CI = 95% confidence interval; a. Included studies had a median follow-up length of 10 years, range 0-25 years. |
| --- |


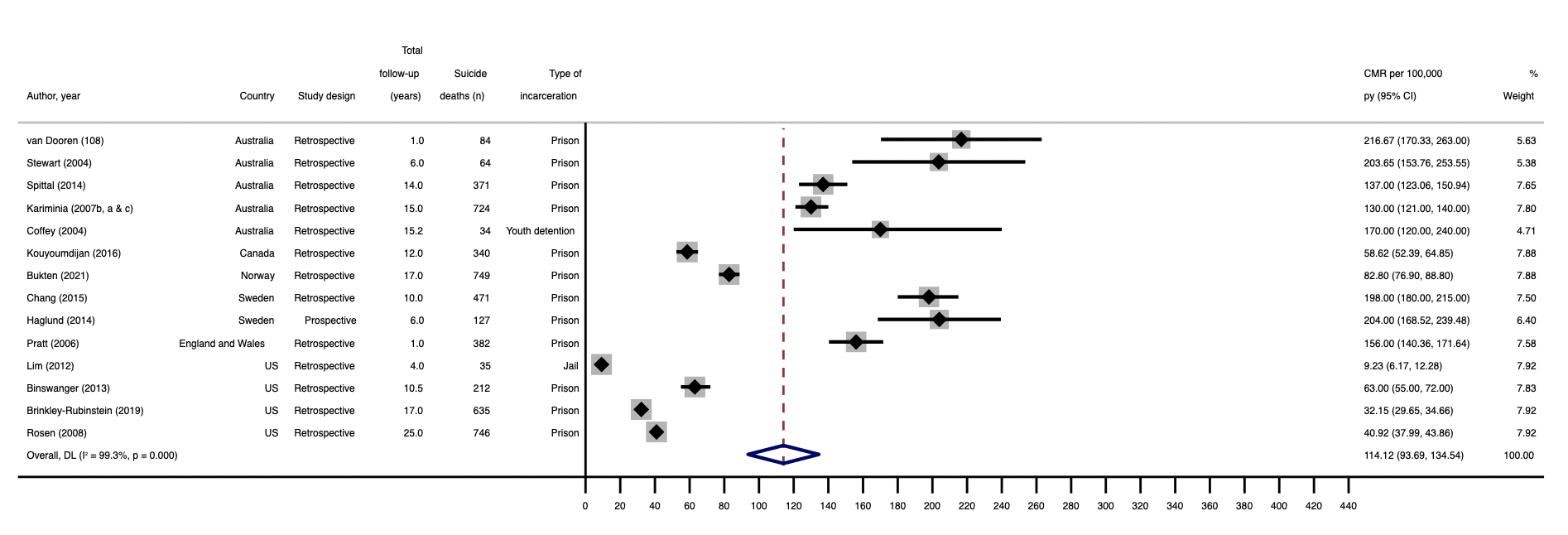


Figure S8. Sensitivity Analysis 2: Non-sex stratified samples pooled CMR, restricting analysis to studies having a score above the median on the MASTER scale

Table S10. Sensitivity analysis 2: Meta-regression for non-sex stratified samples CMR, restricting analysis to studies having a score above the median on the MASTER scale

| **Factor** | **Number of studies** | **CMR (95% CI) per 100,000 person years** | **p-value** | **I^2^** |
| --- | --- | --- | --- | --- |
| **Type of incarceration facility** |  |  |  |  |
| Jail | 1 | 9.23 (0.00, 158.78) | 0.249 | 99.21% |
| Prison | 12 | 124.70 (80.88, 168.52) |  |  |
| Youth detention | 1 | 170 (5.85, 334.15) |  |  |
|  |  |  |  |  |
| **Study design** |  |  |  |  |
| Prospective | 1 | 204.00 (47.15, 360.85) | 0.245 | 99.35% |
| Retrospective | 13 | 112.71 (69.89, 155.53) |  |  |
|  |  |  |  |  |
| **Interval censoring** |  |  |  |  |
| No | 4 | 139.83 (58.79, 220.88) | 0.526 | 99.37% |
| Yes | 10 | 111.14 (60.31, 161.97) |  |  |
|  |  |  |  |  |
| **Total length of follow-up (years)^a^** |  |  |  |  |
| ≤10 years | 6 | 161.83 (104.33, 219.32) | 0.052 | 99.31% |
| >10 years | 8 | 87.23 (38.36, 136.10) |  |  |
|  |  |  |  |  |
| **Country** |  |  |  |  |
| Australia | 5 | 162.13 (128.93, 195.33) | <0.001 | 97.44% |
| Canada | 1 | 58.62 (0.00, 120.09) |  |  |
| Norway | 1 | 82.80 (21.37, 144.23) |  |  |
| Sweden | 2 | 200.58 (151.87, 249.29) |  |  |
| England and Wales | 1 | 156.00 (92.19, 219.81) |  |  |
| US | 4 | 36.17 (5.52, 66.81) |  |  |
|  |  |  |  |  |
| **Male only samples** |  |  |  |  |
| No | 13 | 125.34 (82.13, 168.56) | 0.269 | 99.38% |
| Yes | 1 | 40.92 (0.00, 193.78) |  |  |
| Note: CMR = crude mortality rate; 95%CI = 95% confidence interval; a. Included studies had a median follow-up length of 10 years, range 0-25 years. | | | | |


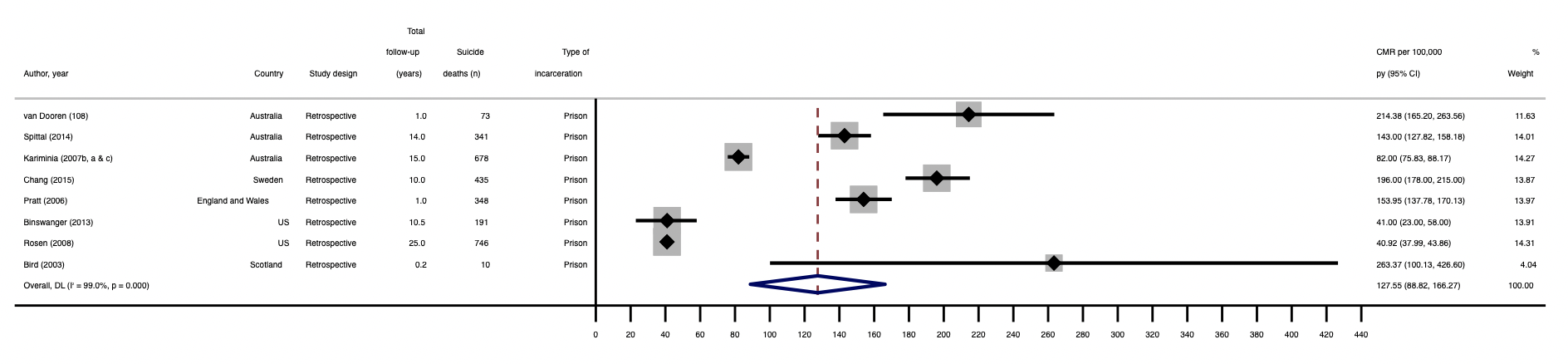


Figure S9. Sensitivity Analysis 3: Male-only pooled CMR, including studies that reported the measures of interest but did not meet the criteria for inclusion

Table S11. Sensitivity analyses for pooled SMRs (non-sex stratified samples only)

|  | **Number of studies** | **Suicide SMR (95%CI)** | **I^2^** |
| --- | --- | --- | --- |
| Non-sex stratified samples SMR |  |  |  |
| Primary analysis | 11 | 7.4 (5.4-9.4) | 98.3 |
| Sensitivity analysis 4: Restricting analysis to studies having a score above the median on the MASTER scale* | 9 | 6.9 (4.7-9.1) | 98.6 |

Note: SMR=standardised mortality ratio. There were no papers on non-sex stratified samples, female-only papers or male-only papers that reported measures of interest but did not meet the criteria for inclusion. There were no female-only papers or male-only papers that included papers with a higher risk of bias. *As there were fewer than 10 included studies, meta-regression was not possible.


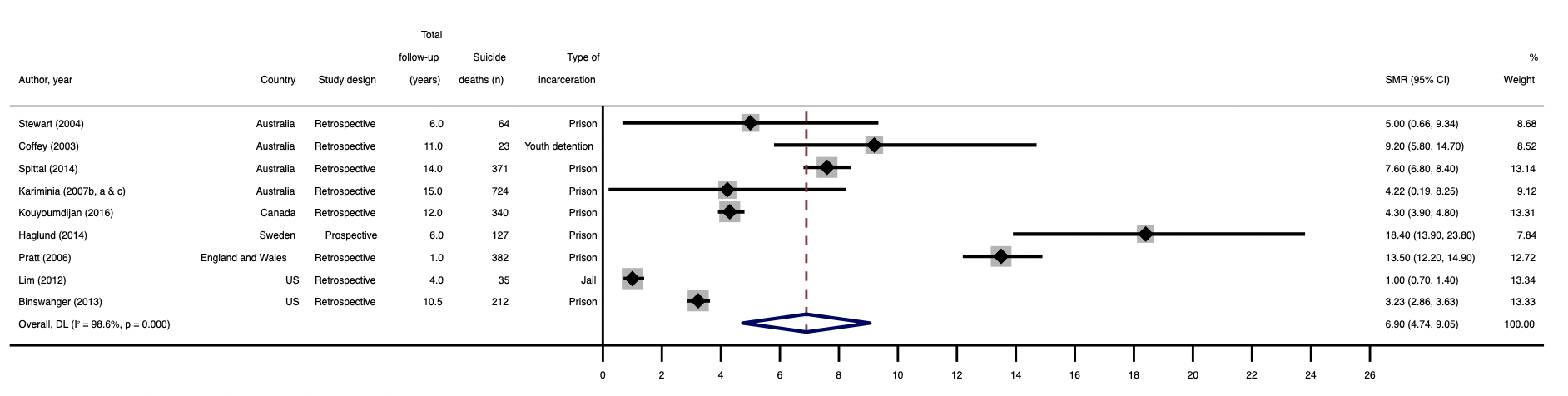
Figure S10. Sensitivity Analysis 4: Non-sex stratified samples pooled SMR, restricting analysis to studies having a score above the median on the MASTER scale

## Supplementary references

1. Barnert ES, Abrams LS, Dudovitz R, Coker TR, Bath E, Tesema L, Nelson BB, Biely C, Chung PJ (2019) What is the relationship between incarceration of children and adult health outcomes? Academic pediatrics 19 (3):342-350

2. Borschmann R, Thomas E, Moran P, Carroll M, Heffernan E, Spittal MJ, Sutherland G, Alati R, Kinner SA (2017) Self-harm following release from prison: A prospective data linkage study. Australian & New Zealand Journal of Psychiatry 51 (3):250-259. doi:10.1177/0004867416640090

3. Borschmann R, Young JT, Moran P, Carroll M, Heffernan E, Spittal M, Mok K, Kinner SA (2017) Ambulance attendances resulting from self-harm after release from prison: a prospective data linkage study. Social Psychiatry and Psychiatric Epidemiology 52 (10):1295-1305

4. Binswanger IA, Stern MF, Deyo RA, Heagerty PJ, Cheadle A, Elmore JG, Koepsell TD (2007) Release from prison - A high risk of death for former inmates. New England Journal of Medicine 356 (2):157-165. doi:10.1056/NEJMsa064115

5. Binswanger IA, Blatchford PJ, Mueller SR, Stern MF (2013) Mortality After Prison Release: Opioid Overdose and Other Causes of Death, Risk Factors, and Time Trends From 1999 to 2009. Annals of internal medicine 159 (9):592-600. doi:10.7326/0003-4819-159-9-201311050-00005

6. Bird SH, Hutchinson SJ (2003) Male drugs-related deaths in the fortnight after release from prison: Scotland, 1996-99. Addiction 98 (2):185-190. doi:<http://dx.doi.org/10.1046/j.1360-0443.2003.00264.x>

7. Brinkley-Rubinstein L, Sivaraman J, Rosen DL, Cloud DH, Junker G, Proescholdbell S, Shanahan ME, Ranapurwala SI (2019) Association of Restrictive Housing during Incarceration with Mortality after Release. JAMA Network Open. doi:<http://dx.doi.org/10.1001/jamanetworkopen.2019.12516>

8. Bukten A, Stavseth MR, Skurtveit S, Tverdal A, Strang J, Clausen T (2017) High risk of overdose death following release from prison: variations in mortality during a 15‐year observation period. Addiction 112 (8):1432-1439

9. Bukten A, Stavseth MR (2021) Suicide in Prison and After Release: A 17-Year National Cohort Study.

10. Chang Z, Lichtenstein P, Larsson H, Fazel S (2015) Substance use disorders, psychiatric disorders, and mortality after release from prison: A nationwide longitudinal cohort study. The Lancet Psychiatry 2 (5):422-430. doi:<http://dx.doi.org/10.1016/S2215-0366%2815%2900088-7>

11. Coffey C, Veit F, Wolfe R, Cini E, Patton GC (2003) Mortality in young offenders: Retrospective cohort study. British Medical Journal 326 (7398):1064-1066

12. Coffey C, Wolfe R, Lovett AW, Moran P, Cini E, Patton GC (2004) Predicting death in young offenders: a retrospective cohort study. The Medical journal of Australia 181 (9):473-477

13. Dirkzwager A, Nieuwbeerta P, Blokland A (2012) Effects of first-time imprisonment on postprison mortality: a 25-year follow-up study with a matched control group. Journal of Research in Crime and Delinquency 49 (3):383-419

14. Farrell M, Marsden J (2008) Acute risk of drug‐related death among newly released prisoners in England and Wales. Addiction 103 (2):251-255

15. Graham A (2003) Post-prison mortality: unnatural death among people released from Victorian prisons between January 1990 and December 1999. Australian & New Zealand journal of criminology 36 (1):94-108

16. Haglund A, Tidemalm D, Jokinen J, Långström N, Liechtenstein P, Fazel S, Runeson B (2014) Suicide after release from prison-a population-based cohort study from Sweden. The Journal of clinical psychiatry 75 (10):1047

17. Harding-Pink D (1990) Mortality following release from prison. Medicine, Science and the Law 30 (1):12-16

18. Jones M, Kearney GD, Xu X, Norwood T, Proescholdbell SK (2017) Mortality Rates and Cause of Death Among Former Prison Inmates in North Carolina. North Carolina medical journal 78 (4):223-229. doi:<http://dx.doi.org/10.18043/ncm.78.4.223>

19. Kariminia A, Law MG, Butler TG, Levy MH, Corben SP, Kaldor JM, Grant L (2007) Suicide risk among recently released prisoners in New South Wales, Australia. AUSTRALASIAN MEDICAL PUBLISHING COMPANY LTD, Australia

20. Kariminia A, Butler TG, Corben SP, Levy MH, Grant L, Kaldor JM, Law MG (2007) Extreme cause-specific mortality in a cohort of adult prisoners--1988 to 2002: a data-linkage study. International Journal Of Epidemiology 36 (2):310-316

21. Kouyoumdjian FG, Kiefer L, Wobeser W, Gonzalez A, Hwang SW (2016) Mortality over 12 years of follow-up in people admitted to provincial custody in Ontario: a retrospective cohort study. CMAJ open 4 (2):E153-161. doi:<https://dx.doi.org/10.9778/cmajo.20150098>

22. Lim S, Seligson AL, Parvez FM, Luther CW, Mavinkurve MP, Binswanger IA, Kerker BD (2012) Risks of drug-related death, suicide, and homicide during the immediate post-release period among people released from New York city jails, 2001-2005. American Journal of Epidemiology 175 (6):519-526. doi:10.1093/aje/kwr327

23. Pratt D, Piper M, Appleby L, Webb R, Shaw J (2006) Suicide in recently released prisoners: a population-based cohort study. Lancet 368 (9530):119-123

24. Rosen DL, Schoenbach VJ, Wohl DA (2008) All-cause and cause-specific mortality among men released from state prison, 1980 - 2005. American Journal Of Public Health 98 (12):2278-2284. doi:10.2105/AJPH.2007.121855

25. Rosen DL, Kavee AL, Brinkley-Rubinstein L (2020) Postrelease mortality among persons hospitalized during their incarceration. Annals of epidemiology 45:54-60

26. Sailas ES, Feodoroff B, Lindberg NC, Virkkunen ME, Sund R, Wahlbeck K (2006) The mortality of young offenders sentenced to prison and its association with psychiatric disorders: a register study. The European Journal of Public Health 16 (2):193-197

27. Spittal MJ, Forsyth S, Pirkis J, Alati R, Kinner SA (2014) Suicide in adults released from prison in Queensland, Australia: a cohort study. Journal Of Epidemiology And Community Health 68 (10):993-998. doi:10.1136/jech-2014-204295

28. Stewart LM, Henderson CJ, Hobbs MST, S. C. Ridout MST, M. W. Knuiman MST (2004) Risk of death in prisoners after release from jail. Australian & New Zealand Journal of Public Health 28 (1):32-36

29. van Dooren K, Kinner SA, Forsyth S (2013) Risk of death for young ex-prisoners in the year following release from adult prison. Australian and New Zealand journal of public health 37 (4):377-382. doi:<http://dx.doi.org/10.1111/1753-6405.12087>
